# The cellular landscape of the normal kidney allograft: Main players balancing the alloimmune response

**DOI:** 10.3389/frtra.2022.988238

**Published:** 2022-10-17

**Authors:** Jennifer M. McDaniels, Amol C. Shetty, Thomas V. Rousselle, Elissa Bardhi, Daniel G. Maluf, Valeria R. Mas

**Affiliations:** ^1^Surgical Sciences Division, Department of Surgery, University of Maryland, Baltimore, MD, United States; ^2^Institute for Genome Sciences, School of Medicine, University of Maryland, Baltimore, MD, United States; ^3^Program in Transplantation, School of Medicine, University of Maryland, Baltimore, MD, United States

**Keywords:** transplantation, alloimmune response, core needle biopsies, single nuclei RNA-sequencing, human kidney

## Abstract

Despite recent advances made in short-term outcomes; minimal improvements have been observed in long-term kidney transplantation outcomes. Due to an imbalance between organ transplant availability and patient waiting list, expanding kidney allograft longevity is a critical need in the field. Prior studies have either focused on early ischemic and immunological conditions affecting kidney allografts (e.g., delayed graft function, acute rejection) or late stage chronic injury when interventions are no longer feasible. However, studies characterizing kidney allografts with normal function by its cellular distribution, cell-cell interactions, and associated molecular pathways are lacking. Herein, we used single nuclei RNA-sequencing to uncover the cellular landscape and transcriptome of the normal kidney allograft. We profiled 40,950 nuclei from seven human kidney biopsies (normal native, *N* = 3; normal allograft, *N* = 4); normal allograft protocol biopsies were collected ≥15-months post-transplant. A total of 17 distinct cell clusters were identified with proximal tubules (25.70 and 21.01%), distal tubules (15.22 and 18.20%), and endothelial cells (EC) (4.26 and 9.94%) constituting the major cell populations of normal native and normal allograft kidneys, respectively. A large proportion of cycling cells from normal native kidneys were in G1-phase (43.96%) whereas cells from normal allograft were predominantly in S-phase (32.69%). This result suggests that transcriptional differences between normal native and normal allograft biopsies are dependent on the new host environment, immunosuppression, and injury-affliction. In the normal allograft, EC-specific genes upregulated metabolism, the immune response, and cellular growth, emphasizing their role in maintaining homeostasis during the ongoing alloreactive stress response. Immune cells, including B (2.81%), macrophages (24.96%), monocytes (15.29%), natural killer (NK) (12.83%), neutrophils (8.44%), and T cells (14.41%, were increased in normal allografts despite lack of histological or clinical evidence of acute rejection. Phenotypic characterization of immune cell markers supported lymphocyte activation and proinflammatory cytokines signaling pathways (i.e., *IL-15, IL-32*). The activation of B, NK, and T cells reveals potential immune cells underlying subclinical inflammation and repair. These single nuclei analyses provide novel insights into kidney and immune cell associated signaling pathways that portray kidney grafts with normal allograft function beyond 2-years post-transplant, revealing a novel perspective in understanding long-term allograft graft survival.

## Introduction

1-year (patient and allograft) survival after kidney transplantation (KT) has progressed, significantly. However, long-term transplant outcomes after 5-years have shown minimal improvements (1–4). Due to an imbalance between organ transplant availability and patient waiting list, expanding kidney allograft longevity is a critical need in the field. The biological mechanisms explaining the lack of correlation between improved short- and unchanged long-term allograft survival after KT are unknown. Late graft loss after KT occurs due to chronic allograft dysfunction (CAD) (5–14), a time-dependent, progressive, and irreversible condition that is often diagnosed late in its course. The origin, functional heterogeneity, differentiation mechanisms, and trajectories of injury-driving cells in the human kidney graft have yet to be discerned, restricting the discovery of therapeutic targets and agents. Over the last decade, transcriptomic profiling has emerged as a powerful approach for revealing unbiased biological information useful for post-transplant management.

Single cell resolution of the human kidney produces large, multidimensional data that empowers researchers to address vast biological questions. Although single nuclei (sn) RNA-seq presents many technical challenges, this robust approach enables the study of complex kidney diseases (e.g., acute and chronic kidney injury), identification of cell-specific injury pathways, and alterations in gene expression within a single cell cluster (15–21). Ultimately, a comprehensive study of kidney-related diseases will drive the success of personalized therapeutic strategies (22, 23).

Single cell transcriptome approaches applied to human kidney allograft samples are in its infancy, with a limited number of reports in the field. Limited published data include the evaluation of pathological conditions affecting the graft compared to normal native kidneys as controls (16, 24–28). Nevertheless, comparative analysis utilizing the native kidney has many known limitations. The native kidney fails to represent the cellular adaptions after immunosuppression or injury infliction, specifically those commonly linked with ischemia reperfusion injury (IRI) proven in human (16, 27) and murine (29) models. Moreover, native kidneys do not represent the influence of the alloimmune response ongoing in the kidney grafts nor the effect of immunosuppression.

In this study, normal native kidneys (*N* = 3) and normal allograft kidneys (*N* = 4) (protocol biopsies with ≥15-months post-transplant) were analyzed to determine the transcriptome of the normal kidney graft at single cell resolution. We tested the hypothesis that discerning the main cellular and molecular players contributing to sustained function in normal kidney grafts will provide further insight on protective pathways and balanced cell-cell interactions that favor the host-recipient co-existence environment. This information may have a critical impact on the identification of new approaches to improve long-term kidney graft outcomes.

A total of 40,950 nuclei (normal native: 12,993 nuclei and normal allograft: 27,957 nuclei) from human kidneys were integrated and 17 major cell clusters were generated. We evaluated differences in epithelial, endothelial, podocytes, and fibroblasts influenced by the alloimmune response (infiltrating and resident immune cells) and chronic immunosuppression exposition. To note, proximal tubule cells derived from normal allografts exhibited a pattern of injury and a senescence phenotype, while also maintaining physiological functions. Endothelial cell heterogeneity was characterized by in-depth integrative analyses. We identified six endothelial sub-clusters in the normal native kidneys and 7 in the normal allografts with minimum gene marker overlap. Two podocyte sub-clusters (POD1-2) also maintained normal functions relating to cell cytoskeleton organization and cell communication or sodium transporter and cadherin binding, respectively, indicating that podocytes play a minor role in propagation of injury. Lastly, we described that the immune cell landscape of the normal allograft showed six sub-clusters of B, macrophages, natural killer, neutrophils, and T cells. The alloimmune response in the functional graft was characterized by enrichment in leukocyte cell adhesion, cytokine production, and T cell activation, resulting in upregulation of proinflammatory signals. Concluding, an ongoing silent transcriptional inflammatory background in the normal allografts after ≥15-months post-transplant was detected, indicating that despite clinical evident damage, these kidneys are continuously dealing with balancing low level of injury and physiological repair. Further understanding of these cell-specific transcriptional responses are critical for developing of strategies sought to maintain and restore kidney allograft function.

## Results

### Patient samples

**Figure 1:**
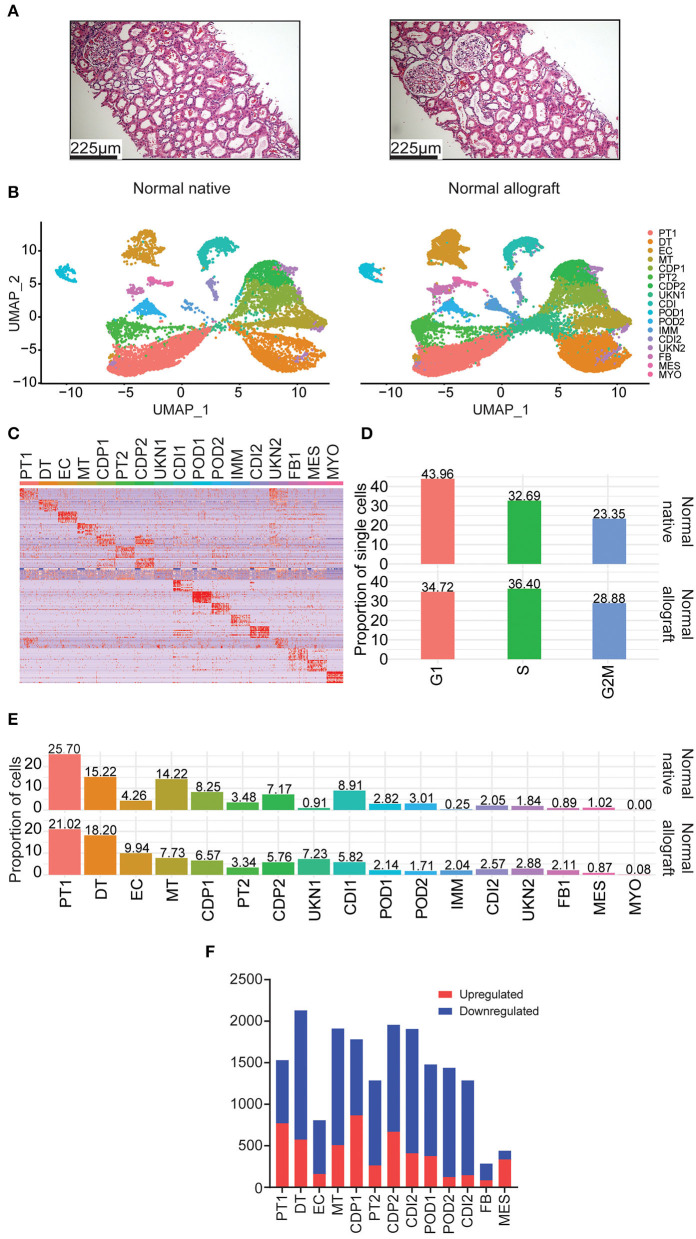
Single nuclei RNA sequencing of human kidney biopsies. **(A)** Representative kidney allograft images with Hematoxylin and Eosin (H&E) staining showing architecturally normal kidney parenchyma. Scale bars, 225 mm. **(B)** UMAP integration of nuclei clustered into 17 distinct cell types from normal native kidney biopsies (N = 3) and normal allograft kidney biopsies (N = 4). **(C)** Heatmap of cell markers used to define each cell cluster using the Human CellMarker Database. PT1, proximal tubule cells 1; DT1, distal tubule cells 1; EC, endothelial cells; MT, mixed tubule cells; CDP1, collecting duct principal cell 1; PT2, proximal tubule cells 2; CDP2, collecting duct principal 2; UKN1, unknown 1; CDI, collecting duct intercalating cells 1; POD1, podocyte 1; POD2, podocyte 2; IMM, immune cells; CDI2, collecting duct intercalating cells 2; UKN2, unknown 2; FB, fibroblasts; MES, mesangial cells; and MYO, myocyte. **(D)** Comparison of cell cycle states between normal native and normal allograft kidney biopsies. Orange, G1; green, S; blue, G2M. **(E)** Distribution of cell type populations identified in each sample. **(F)** Distribution of differentially expressed genes (DEGs) that are up- (red) or down-regulated (blue) in the normal allograft relative to normal native biopsies.

In this study, normal native kidneys [*N* = 3, GSE131882 (24, 25)] and normal allograft kidneys (*N* = 4) (protocol biopsies) were evaluated to determine the transcriptome of the normal kidney graft at single cell resolution. Demographic characteristics and histological evaluation based on Banff scoring system (30, 31) are shown in Tables 1, 2 and Supplementary Table S1. Mean time post-transplant from normal allografts at the time of biopsy collection was: 18-months and mean creatinine values: 0.89 mg/dL. Mean donor age value between groups (native vs. normal allograft) was: 59 and 27.5 years old, respectively. However, the mean kidney transplant recipient age value was 51 years old. At 36-months post-transplant, three out of four kidney allografts maintained normal function (eGFR >60 mL/min/ 1.73 m^2^) and one out of four patients showed normal function at 58-months post-transplant (with a longer follow-up). All patients received triple immunosuppression with calcineurin inhibitors, mycophenolate mofetil, and steroids. The native biopsies did not have evidence of glomerulosclerosis, interstitial fibrosis, or immune cell infiltrate (24, 25). Likewise, the normal graft biopsies, which were analyzed by two independent pathologists (Figure 1A), did not show signs of acute rejection, BK virus or ongoing injury as reflected by Banff scores.

**Table 1:** Patient samples demographics and pathologic characteristics.

**Sample ID**		**Donor characteristics**						**Recipient characteristics**					
		**Age**	**Sex**	**sCR**	**Glomerulo-sclerosis**	**Arterio-sclerosis**	**IFTA**	**Age**	**Sex**	**sCR**	**Glomerulo-sclerosis**	**Arterio-sclerosis**	**IFTA**
Normal native^*^	GSM3823939	54	M	1.28	None (<10%)	Moderate	1-10%	-	-	-	-	-	-
	GSM3823940	62	M	1.21	None (<10%)	Moderate	1–10%	-	-	-	-	-	-
	GSM3823941	61	F	0.89	None (<10%)	Mild	1–10%	-	-	-	-	-	-
Normal allograft	NA1	21	M	-	None (<10%)	None	0%	70	M	0.80	None (<10%)	None	0%
	NA2	49	M	-	None (<10%)	None	0%	56	F	1.00	None (<10%)	None	0%
	NA3	17	M	-	None (<10%)	None	0%	32	F	0.76	None (<10%)	None	0%
	NA4	23	F	-	None (<10%)	None	0%	46	F	1.00	None (<10%)	None	0%

* Samples were previously published (24, 25).

Age is denoted in years; sCR, serum creatinine levels denoted in mg/dL.

**Table 2:** Normal kidney allograft Banff classifications.

**Category**	**NA1**	**NA2**	**NA3**	**NA4**
Interstitial inflammation (i)	0	0	0	0
Tubulitis (t)	0	0	0	0
Glomerulitis (g)	0	0	0	0
Peritubular capillaritis (ptc)	0	0	0	0
Intimal arteritis (v)	0	0	0	0
Interstitial fibrosis (ci)	0	0	0	0
Tubular atrophy (ct)	0	0	0	1
GBM double contours (cg)	0	0	0	0
PTC multilayering (ptcml)	0	0	0	0
Vascular fibrous intimal thickening (cv)	0	0	0	0
Mesangial matrix expansion (mm)	1	0	0	0
Arteriolar hyalinosis (ah)	0	0	0	0
Inflammation in the area of IFTA (i-IFTA)	0	0	0	0

All biopsies were assessed using light (LM), immunofluoresce (IF), and electron microscopy (EM) and reported independently by two transplant pathologists using Banff 2019 (30) update of the Banff 97 (31) classification. All biopsies were negative for peritubular capillary staining for complement factor 4 fragment d (C4d) tested by immunofluorescence. All biopsies were negative for BK virus (SV40 large T antigen, immunoperoxidase staining).

### Quality control

To ensure that the datasets could be combined and assessed in our analysis, stringent quality controls were met. There was no significant difference in sequencing quality control among the samples evaluated by the number of RNA features, RNA reads, and percentage of mitochondrial contribution (Supplementary Figure S1).

### Main cell populations

Using the normal allograft kidney biopsies, a total of 27,957 nuclei were evaluated. The samples were first visualized using uniform manifold approximation and projection (UMAP). A total of 17 main cell type clusters were generated (Figures 1B,C). Absolute number of cells per cluster are listed in Table 3. Individual UMAP per samples and cell proportions are shown in Supplementary Figure S2. The Human CellMarker Database (32) was used to call cell types based on the differential expression of marker genes detected in each cluster (Figure 1C). These clusters included two proximal tubule cells (PT1-2), distal tubule cells (DT), endothelial cells (ECs), mixed tubule (MT), two collecting duct proximal cells (CDP1-2), two podocyte clusters (POD1-2), immune cells (IMM), two collecting duct intermediate clusters (CDI1-2), fibroblasts (FB), mesangial (MES), and myocytes (MYO). Two clusters of unknown cells were identified (UNK1-2). Evaluation of the underlying cell state of replication also highlighted significant transcriptomics changes among cell clusters. The proportion of cycling cells were more abundant in the G1-phase (43.96%) for the native kidney whereas the S-phase were more abundant in the normal allograft kidney (Figure 1D). The analysis estimated the percentages of cycling cells in G1, S, and G2M-phases as 43.96, 32.69, and 23.35% for normal native and 34.72, 36.40, and 28.88% for normal allograft (Figure 1D).

**Table 3:** Total number and proportion of single nuclei per cell type cluster.

		**Total number of cells**			**Proportion of total cells (%)**		
	**Cell cluster**	**Normal native**	**Normal allograft**	**Total**	**Normal native**	**Normal allograft**	**Total**
1	Proximal tubule 1 (PT1)	3,339	5,877	9,216	25.7	21.02	46.72
2	Distal tubule (DT)	1,978	5,089	7,067	15.22	18.2	33.42
3	Endothelial (EC)	554	2,778	3,332	4.26	9.94	14.20
4	Mixed tubule (MT)	1,847	2,160	4,007	14.22	7.73	21.94
5	Collecting duct principal 1 (CDP1)	1,072	1,836	2,908	8.25	6.57	14.82
6	Proximal tubule 2 (PT2)	452	935	1,387	3.48	3.34	6.82
7	Collecting duct principal 2 (CDP2)	932	1,610	2,542	7.17	5.76	12.93
8	Unknown 1 (UKN1)	118	2,021	2,139	0.91	7.23	8.14
9	Collecting duct intercalated 1 (CDI1)	1,158	1,628	2,786	8.91	5.82	14.74
10	Podocyte 1 (POD1)	366	598	964	2.82	2.14	4.96
11	Podocyte 2 (POD2)	391	479	870	3.01	1.71	4.72
12	Immune (IMM)	33	569	602	0.25	2.04	2.29
13	Collecting duct intercalated 2 (CDI2)	266	719	985	2.05	2.57	4.62
14	Unknown 2 (UKN2)	239	804	1,043	1.84	2.88	4.72
15	Fibroblast (FB)	116	589	705	0.89	2.11	3.00
16	Mesangial (MES)	132	244	376	1.02	0.87	1.89
17	Myocytes (MYO)	0	21	21	0.00	0.08	0.08
	Total	12,993	27,957	40,950	100	100	100

### Non-immune cell type differences amongst the groups

Cell proportions were used to report major differences between groups. The most abundant cell type was the PT1 cluster (25.70 and 21.02% for normal native and normal allograft biopsies, respectively) (Figure 1E), which were slightly decreased in normal allografts. Native kidneys were enriched in MT, CDP1-2, CDI1, and POD2 cells. The proportion of MES cells was slightly more abundant in native kidneys (0.89 vs. 1.02%) (Figure 1E). The normal allograft kidney landscape was enriched with a higher proportion of DT, EC, FB, and UNK1-2 when compared to the native kidneys. Notably, EC were approximately 2.3-fold higher in normal allografts compared to native kidneys (9.94 vs. 4.26%, respectively) (Figure 1E). There was an approximate 7-fold increase in cells belonging to the UKN1 cluster (7.23 vs. 0.91%, respectively) (Figure 1E). The number of FB were also increased in the normal allograft kidney (2.11%) compared to normal native kidney (0.89%) (Figure 1E). These broad changes in cell proportion indicated a different cellular composition of the native and normal allograft kidneys likely associated with differential biological functions/pathways defining the two “normal” kidney conditions. Indeed, further exploration of differentially expressed genes (DEGs) identified several downregulated genes in the normal allograft (Figure 1F). Specifically, 71% of the total number of DEGs were downregulated in almost all the parenchymal cells contributing to the normal allograft (excluding PT1), indicating significant differences in the kidney graft transcriptome.

We aimed to elucidate the cell identity of the UNK clusters. UKN1 expressed shared markers with PT1 cluster, including *PRODH2, SORCS1, MIOX, SLC34A1, SLC22A6*, and *CYP4A11* (Supplementary Figure S3). UKN2 also expressed shared PT1 markers but were discrete from UKN1. These markers included *HNF4A-AS, DRAIC, AFM*, and *FMO5*. UKN2 expressed unique markers, *SLC12A1* and *UMOD*, related to Loop of Henle cells (33). Compared to UKN1, UKN2 is a more diverse cluster that shared many expressed markers with other renal clusters (Figure 1C).

Next, the PT sub-clusters were evaluated, PT1 and PT2, together with a mixed tubule subcluster, MT1, to uncover further transcriptional differences. The number of upregulated DEGs for these clusters were 771, 263, 507 whereas the number of downregulated DEGs were 759, 1,023, 1,403 for PT1, PT2, and MT1, respectively. Although these three clusters shared 10.4% (112) of the number of upregulated DEGs, the PT1 cluster distinctively expressed 42.2% (455) DEGs, contributing mostly to normal metabolic pathways (Table 4, Supplementary Table S2). PT1 cells were characterized by presenting “anchor” genes involved in normal cell function and identity (*LRP2, SLC22A6, DPYS, AFM, SLC5A12, AK4, SLC4A4, KHK, GHR, SLC22A12, HNF4A*, and *SLC39A5*). PT2 and MT cells presented PT1 markers and shared genes that were enriched in adherens junction, focal adhesion, and actin cytoskeleton regulation (Table 4). Interestingly, evaluating upregulated DEGs in normal allografts compared to native kidneys across PT1, PT2, and MT clusters presented a signature of cellular senescence distinct to the normal allografts (Table 4, Supplementary Table S3). Moreover, longevity regulation associated genes were downregulated in PT2 and MT cell clusters (Table 5). DEG analyses of epithelial cell clusters between the study groups showed that PT1 cells presented upregulation of metabolic pathways, actin cytoskeleton, and focal adhesion (Table 4) and downregulation of AMPK and EGFR tyrosine kinase inhibitor resistance signaling pathway (Table 5). Of which, metabolism is a vital function of PT cells (21). Critically, these cells were decreased in normal allografts compared to native kidneys (Figure 1E). Upregulated pathways associated with PT2 in the normal allografts were enriched in regulation of mesenchymal cell apoptotic process involved in metanephros development, SLC-mediated transmembrane transport, and VEGFA-VEGFR2 signaling (Table 4). Moreover, pathways involved in longevity regulation, TNF signaling, mineral absorption and choline metabolism were downregulated in PT2 (Table 5). MT cells were characterized by upregulation of adherens junction interactions, focal adhesion, VEGFA-VEGFR2 signaling (Table 4). Unique downregulated pathways included ubiquitin-mediated proteolysis, ErbB signaling, and MAPK signaling (Table 5).

**Table 4:** DAVID enrichment analysis of KEGG upregulated pathways of tubule cells derived from normal allografts compared to normal native biopsies.

**Enrichment pathway**	**DEGs**	***p*-value**
	**Upregulated in PT1**	
Metabolic pathways	*AGPAT3 HIBCH ABAT PHYKPL ACLY DNMT3A DGLUCY FGGY GMDS MECOM NDUFV3 NME7 ACO2 ACOX2 ACYP2 ADK AK2 ALDH2 ALDH6A1 ALDH7A1 ALDH8A1 AGPS ACY1 ACMSD AFMID ARSB ASPA B4GALT5 BHMT2 BHMT3 BTD CA12 CERS4 CERS6 CHDH DGKB DGKH DPYS ENTPD5 ENOSF1 EHMT1 FMO1 FMO5 FBP1 FUT6 GALM BBOX1 GCNT2 GATM HEXA HLCS HAO2 IMPA2 INPP5B KMO MAN1C1 MTAP MTMR3 NSD1 OGDH PNPLA3 PIK3CB PIP5K1A PDE10A PDE1A PDE7B PLCB1 PLCG2 PLD1 PLPP1 GALNT11 GALNT14 GALNT18 P4HA2 PDXK SHPK SPTLC3 SORD SGPP1 UPP2 XYLB*	6.5E-4
Focal adhesion	*RAPGEF1 ARHGAP35 ACTN4 COL4A2 DOCK1 EGF FLNB GRB2 ITGB4 ITGB8 LAMB1 MYLK PIK3CB PIP5K1A PRKCA PTK2 VEGFA VAV2 VAV3*	4.7E-4
Actin cytoskeleton regulation	*ARHGAP35 ACTR3C ACTN4 DOCK1 EGF EZR ITGB5 ITGB8 MYH9 MYLK PIK3CB PIP5K1A PTK2 SSH2 SPATA13 VAV2 VAV3*	7.1E-3
Adherens junction interactions	*CDH6 CDH9 CTNNA1 CTNND1 JUP*	3.5E-2
Cellular senescence	*RAD9A MAPK14 TRPM7*	7.6E-2^*^
	**Upregulated in PT2**	
Regulation of mesenchymal cell apoptotic process in metanephros development	*HNF1B PAX2 PAX8*	2.1e-4
SLC-mediated transmembrane transport	*MFSD4B SLC1A1 SLC12A1 SLC12A3 SLAC13A1 SLC2A11 SLC8A1 SLC30A8 SLC44A3 SLC6A6 SLC8A1*	2.9E-4
Adherens junction interactions	*CDH6 CDH9 CTNND1 JUP*	4.9E-3
Cellular senescence	*RAD50 HIPK2 NFATC3 PPP3R1 SQSTM1*	7.2E-2
Actin cytoskeleton regulation	*ARHGAP35 DOCK1 EGF SSH2 SPATA13 VAV3*	6.8E-2
VEGFA-VEGFR2 signaling	*CTNNBD1 DOCK1 JUP SHB VAV3*	2.0E-2
	**Upregulated in MT**	
Adherens junction interactions	*LMO7 SMAD2 ACTN4 CTNND1 FGFR1 IGF1R PTPRF PTPRJ PTPRM*	3.3E-4
Focal adhesion	*BCL2 ARHGAP35 ACTN4 CAPN2 COL4A1 COL4A2 FLNB IGF1R LAMB1 PAK4 PIP5K1A PRKCA TLN2 VEGFA VAV2*	4.4E-4
Gastric acid secretion	*ADCY5 CALM2 CA22 KCNJ1 KCNJ10 KCNJ15 KCNJ16 KCNQ1 PRKCA*	5.3E-4
Cellular senescence	*RAD50 RAD9A SMAD3 CALM2 CAPN2 HIPK2 NFATC3 PPPER1 SQSTM1*	3.9E-2
Actin cytoskeleton regulation	*ARHGAP35 ACTN4 BDKRB2 CYF1P2 FGFR1 KNG1 MYH9 PAK4 PIP5K1A SSH1 SPATA13 VAV2*	1.9E-2
VEGFA-VEGFR2 signaling	*SHB CALM2 CTNND1 CYF1P2 JUP PRKCA PRKCZ VEGFA VAV2*	1.5E-3

*N ot significant.

**Table 5:** DAVID enrichment analysis of KEGG downregulated pathways of tubule cells derived from normal allografts compared to normal native biopsies.

**Enrichment pathway**	**DEGs**	***p*-value**
	**Downregulated in PT1**	
AMPK signaling	*ACACB AKT3 FOXO1 FOXO3 HNF4A PIK3CB PFKL PFKFB2 PPP2CB PPP2R2A PPP2R2B PRKAA2 RPTOR*	1.40E-03
EGFR tyrosine kinase inhibitor resistance	*AKT3 ERBB3 FOXO3 IL6R NRG1 PDGFC PIK3CB PLCG1 PRKCA SOS2*	2.30E-03
Biosynthesis of amino acids	*ABHD14A-ACY1 ACO1 ALDOB BCAT2 IDH1 MTR PAH PFKL*	5.90E-03
Pantothenate and CoA biosynthesis	*ALDH3A2 BCAT2 DPYD ENPP3 UPB1*	6.50E-03
Tight junction	*AFDN DLG2 EZR MAP3K5 MPDZ PATJ PPP2CB PPP2R2A PPP2R2B PRKAA2 RAPGEF6 RDX YBX3*	2.20E-02
	**Downregulated in PT2**	
Longevity regulation	*AKT3 ATF2 ATF4 CREB5 FOXO1 FOXO3 PIK3CA PIK3R1 PRKAB1 RHEB SESN1 SOD2*	1.60E-04
TNF signaling	*AKT3 ATF2 ATF4 CREB5 DNM1L IL15 JUN MAP2K4 NFKBIA PIK3CA PIK3R1 RPS6KA5 VCAM1*	3.10E-04
Mineral absorption	*ATP1A1 ATP2B1 FTH1 MT1E MT1G MT1H MT1X MT2A SLC26A3*	7.50E-04
Choline metabolism	*AKT3 DGKH1 HIF1A JUN PIK3CA PIK3R1 PLCG1 RHEB SLC22A4 SLC22A5 SOS2*	1.40E-03
	**Downregulated in MT**	
Longevity regulation	*AKT3 ATF2 ATG5 FOXO3 IRS2 KL KRAS NFKB1 PIK3CA PRKAA1 PRKAA2 PRKAB1 PRKACB RB1CC1 RHEB SENS1*	2.50E-04
Ubiquitin-mediated proteolysis	*BIRC6 BRCA1 CBLB CDC27 CUL3 COP1 FBXW11 FBX7 FBXW8 FANCL MDM2 NEDD4L PIAS2 RCHY1 SMURF2 TRIP12 UBA6 UBE2D3 UBE2N UBR5 WWP1*	3.10E-04
ErbB signaling	*AKT3 BRAF CBLB CAMK2D EGF ERBB4 GAB1 KRAS GSK3B MAP2K4 PAK1 PIK3CA PLCG1 PTK2 SOS1*	5.10E-04
MAPK signaling	*BRAF MAPKAPK5 RAPGEF2 ATF2 MAP2K4 MAP2K6 MAP3K2 MAP3K7 MAP4K3 MAP4K5 MEF2A MEF2C NFKB1 PAK1*	8.80E-04
Endocrine and other factor regulated calcium absorption	*AP2B1 ATP1A1 ATP1B1 ATP1B3 ATP2B1 DNM3 GNAQ GNAS KL PRKACB VDR*	1.10E-03

**Figure 2:**
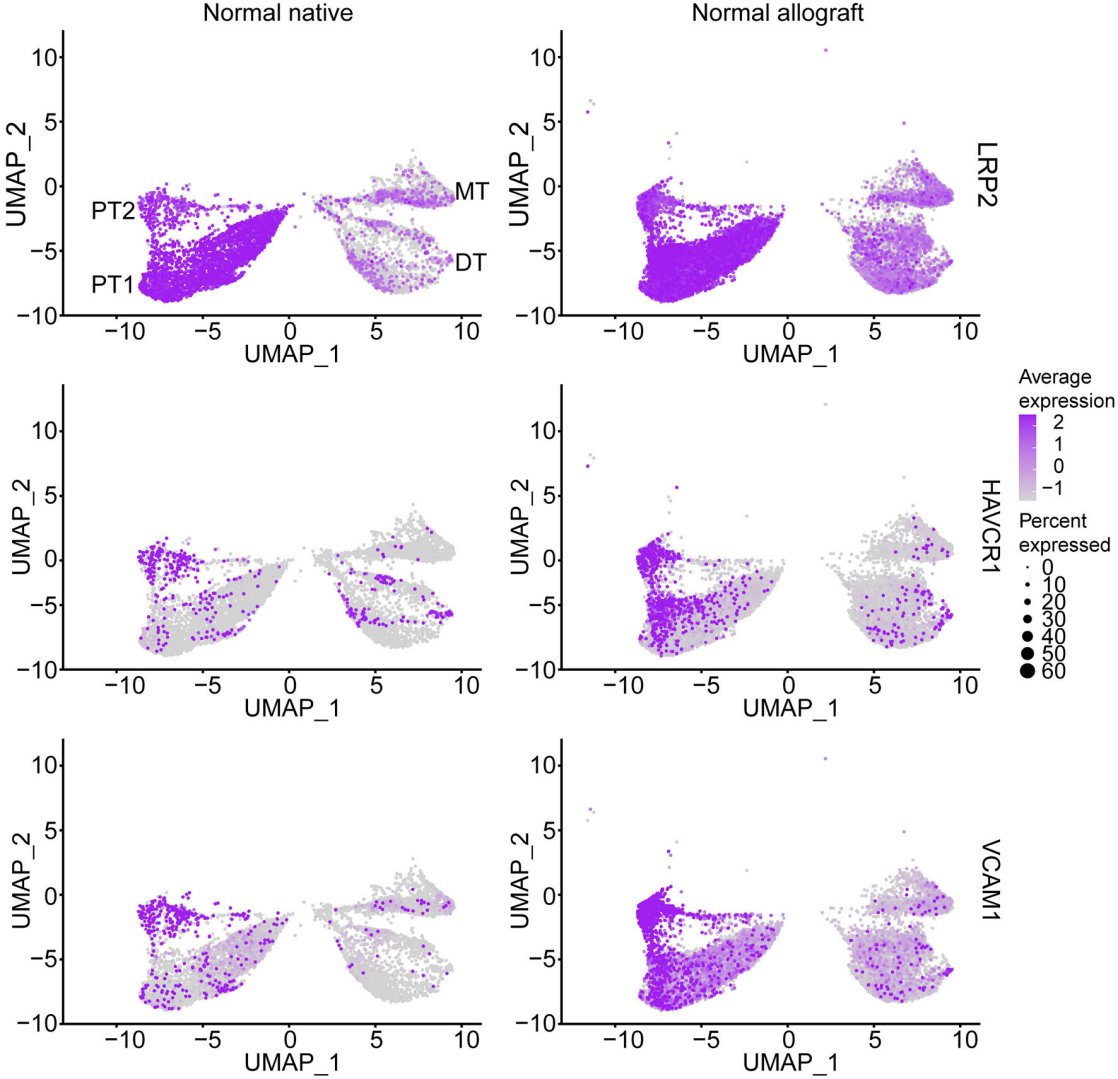
Epithelial tubule cell-specific expression. UMAP visualization of normal proximal cell expression (LRP2) and injury (HAVCR1 and VCAM1) markers. DT, distal tubule cells; PT1, proximal tubule cells 1; PT2, proximal tubule cells 2; and MT, mixed tubule cells. Average expression, Log2 fold change.

As multiple immunological (i.e., acute rejection, IRI) and non-immunological (i.e., chronic immunosuppressive therapy, donor quality) factors are known to cause injury to the graft (34–40), we sought to determine the level of injury inflicted to the proximal tubule cells, as these cells are the first responders to injury (17, 41). Analysis of proximal tubule cell marker gene, *LRP2*, also showed co-expression of *HAVCR1* and *VCAM1* in the PT clusters being more prominent in PT2 (Figure 2), suggesting a higher level of injury in the PT cluster of normal allografts compared to native biopsies (17, 18, 42).

**Figure 3:**
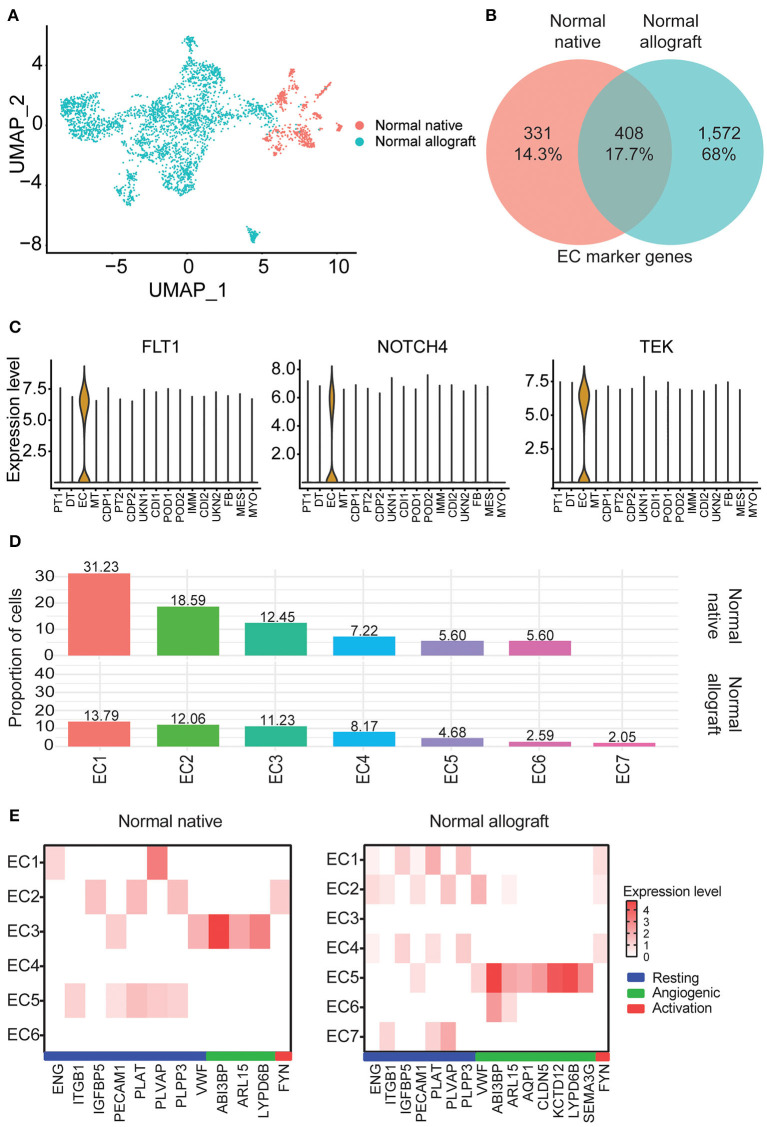
Endothelial cell heterogeneity. **(A)** UMAP visualization of the compiled normal native and allograft biopsies clustered separately. **(B)** Venn diagram of shared endothelial markers. **(C)** Select shared cell markers of the EC subclusters. **(D)** Distribution of endothelial cell (EC) subclusters EC1-7. Cellular contributions of immune and tubule doublets are not shown. **(E)** Heatmap profiling EC states (resting, angiogenic, and activation) by gene expression. Each column represents a gene marker and each row corresponds to cluster number. Expression level, Log2 fold change; blue bar, resting state; green bar, angiogenic state; red bar, activation state.

Kidney transplantation entails a high likelihood of endothelial cell (EC) injury. The endothelium is a target of choice for injury by ischemia-reperfusion, alloantibodies, and autoantibodies (43, 44). EC characterization in the kidney graft with normal function is critical. Notably, EC heterogeneity was observed between the two study groups. Interestingly, these clusters were primarily non-overlapping between groups (Figure 3A). Less than 18% of marker genes were shared between the normal native and allograft groups (Figure 3B) including canonical markers *FLT1, NOTCH4*, and *TEK* (Figure 3C). A total of 7 sub-clusters (EC1-7) were identified in the normal allograft (*N* = 1,530 total cells) whereas 6 sub-clusters (EC1-6) were identified in the normal native (*N* = 292 total cells) group; cell proportions are also shown (Figure 1C). Cluster-specific marker genes are shown in Supplementary Figures S4, S5 and listed in Supplementary Tables S4, S5. EC7, unique for normal kidney allograft samples, highly expressed *VCAN* (Supplementary Figure S5), known to regulate inflammation (45).

ECs are multifunctional involved in angiogenesis, cell proliferation and migration, and vascular homeostasis (46–48). From enrichment pathway and gene ontology analyses of upregulated DEGs, regulation of cell adhesion, VEGFA signaling, tube morphogenesis, and hemostasis were all shared between the groups, although the listed terms and pathways were more significant in the normal allograft group (Supplementary Figures S6A,B). Cell morphogenesis, kinase activity, and positive regulation of cell migration were specific to the normal allograft (Supplementary Figure S6B). Moreover, endothelial states were established using previously published markers (16). For the normal native biopsies, gene signatures were lowly expressed across the 6 clusters. Resting states were associated with EC5 (*ITGB*1^+^, *PECAM*1^+^, *PLAT*^+^, *PLVAP*^+^, *PLPP3*^+^) and the angiogenic state was associated with EC3 (*ABI*3*BP*^+^, *ARL15*^+^, *LYPD6B*^+^*)* clusters (Figure 3E). EC2 expressed *FYN* (Figure 3E), which is one activation-specific gene signature. For normal allograft biopsies, the resting states was associated with EC1 (*ENG*^+^, *IGFBP*5^+^, *PECAM*1^+^, *PLAT*^+^, *PLVAP*9^+^, and *PLPP3*^+^*)* and 2 (*ENG*^+^, *ITGB*1^+^, *PECAM*1^+^, *PLVAP*9^+^, and *VWF*^+^) (Figure 3E). EC5 presented angiogenic properties (*ABI*3*BP*^+^, *ARL15*^+^, *AQP*1^+^, *CLDN*5^+^, *KCD*12^+^, *LYPD*6*B*^+^, *SEMA*3*G*^+^, *SERPINE*2^+^, *VWF*^+^) (Figure 3E). A strong gene signature for activation was not detected, although *FYN* was expressed in EC1, 2, and 4 (Figure 3E). Moreover, the EC7 cluster which is only present in kidney grafts expressed *TLR4*, a protein involved involved in the recruitment of native immune cells (Supplementary Figure S1).

**Figure 4:**
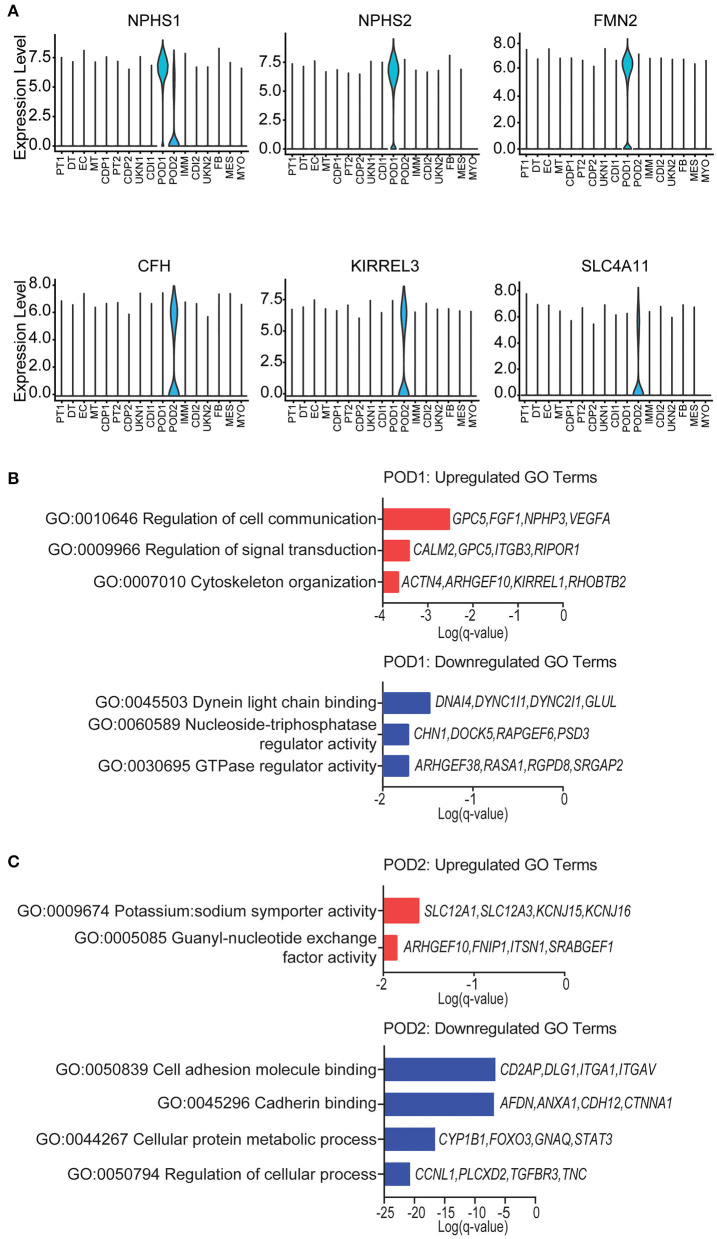
Podocyte identity and enrichment analysis. **(A)** Violin plots of maker gene expression levels associated with each cluster. Expression level, Log2 fold change. **(B)** POD1 top enriched Gene Ontology (GO) terms of (top) upregulated and (bottom) downregulated differentially expressed genes (DEGs). **(C)** POD2 top enriched GO terms of (top) upregulated and (bottom) downregulated gene signatures for immune cells. Select genes are listed.

Podocyte loss has been reported immediately after transplantation in normal allografts (49–52). Therefore, we evaluated the transcriptional differences of podocytes in the normal native and normal allograft samples. Interestingly, we identified 2 distinct podocyte clusters (POD1-2) (Figures 1B,C, 4A), although both clusters positively expressed *nephrin, NPHS1* (Figure 4A). Compared to native kidneys, the normal allografts displayed a 0.68 and 1.30% reduction in POD1-2, respectively (Figure 1E). The results of GO analysis revealed that upregulated DEGs associated with POD1 were significantly enriched in cytoskeleton organization and cell communication whereas downregulated DEGs were enriched in GTPase regulator activity and dynein light chain binding (Figure 4B). For POD2, upregulated DEGs were significantly enriched in symporter activity whereas downregulated DEGs were enriched in cell adhesion and cadherin binding (Figure 4C).

**Figure 5:**
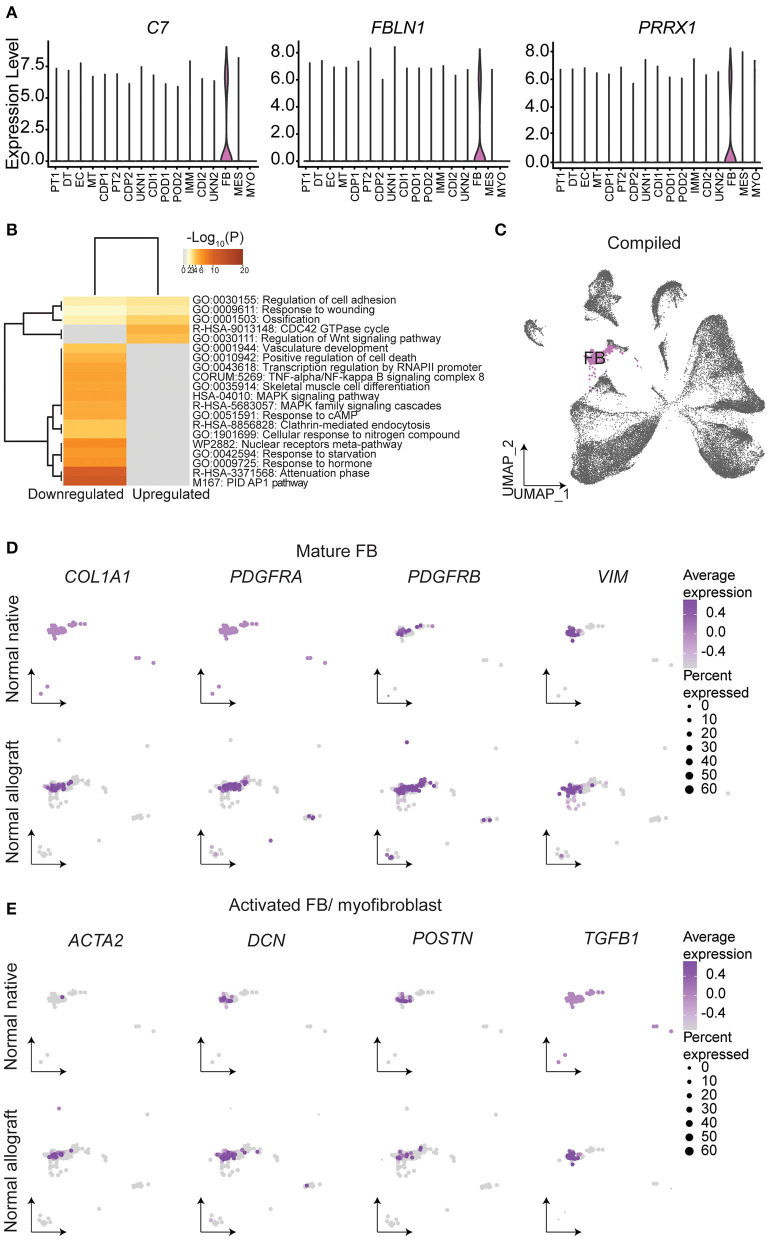
Fibroblast identities and gene signatures. **(A)** Expression of select fibroblast (FB) gene markers. **(B)** Metascape analysis of up- and down-regulated enriched terms in the normal allograft relative to normal native kidneys. **(C)** Compiled UMAP showing spatial location of FB emphasized in **(D)** and **(E)**. Feature UMAP plots of FB expression distribution of **(D)** mature FB and **(E)** activated FB gene signatures. Average expression, Log2 fold change.

Fibroblasts (FB) are known to play a critical role in managing kidney injury and wound healing (53). Critically, the evaluation of cell proportions showed about a 2.4-fold increase in FB (*ADH1B, C7, FBLN1, PRRX1*, and *ADH1B)* (Figure 5A) in normal allograft compared to native kidney biopsies (Figure 1E). Interestingly, *PRRX1* has been recently reported as a master regulator of the fibroblast to myofibroblast transition (53, 54). Also, 84 DEGs were upregulated and 200 DEGs were downregulated in FB cluster derived from normal allografts relative to native normal biopsies (Figure 1E). By performing GO and pathway analysis of DEGs, downregulated pathways included attenuation, positive regulation of cell death, MAPK signaling, and TNFα /NFkβ signaling (Figure 5B). The CDC42 GTPase cycle and Wnt signaling were amongst the upregulated pathways (Figure 5B). CDC42 is an important regulator of the actin cytoskeleton, fibroblast motility, and epithelial wound healing (54–56). Enrichment analysis also identified cell adhesion and response to wound healing as significant enriched pathways (Figure 5B). To determine the role of FB in the wound healing and injury, we determined the cellular state by gene expression visualized on the UMAP (Figures 5C–E). Compared to the normal native group, the normal allograft uniquely expressed both *COL1A1* (adjusted *p-*value = 2.8E-06, Log_2_ fold change (FC): 6.47) and *PDGFRA* (adjusted *p*-value = 5.6E-13, Log_2_ FC: 7.97) while also showing relatively higher expression of *PDGFRB* and *VIM* which confirmed the presence of mature FB (Figure 5D). Interesting, gene expression in the normal allograft also supported the presence of activated FB and/ or myofibroblasts, expressing *ACTA2, DCN*, and *POSTN* (Figure 5D). The activated signature was present in ~20–30% of total FBs and expression levels did not exceed a Log_2_ FC value of 0.4. Thus, a subpopulation of FBs in the normal allograft were distinctly different from normal native biopsies presenting some level of activation and an incipient profibrogenic signature (ECM glycoproteins: *ECM2, IGFBP5*; collagen: *COL1A1*; ECM-affiliated protein: *GPC5*; and a secreted factor: *IGF1*). The expression of COL1A1 was validated at the protein level using Imaging Mass Cytometry (IMC) (Supplementary Figure S7).

### Immune cell differences amongst the groups

**Figure 6:**
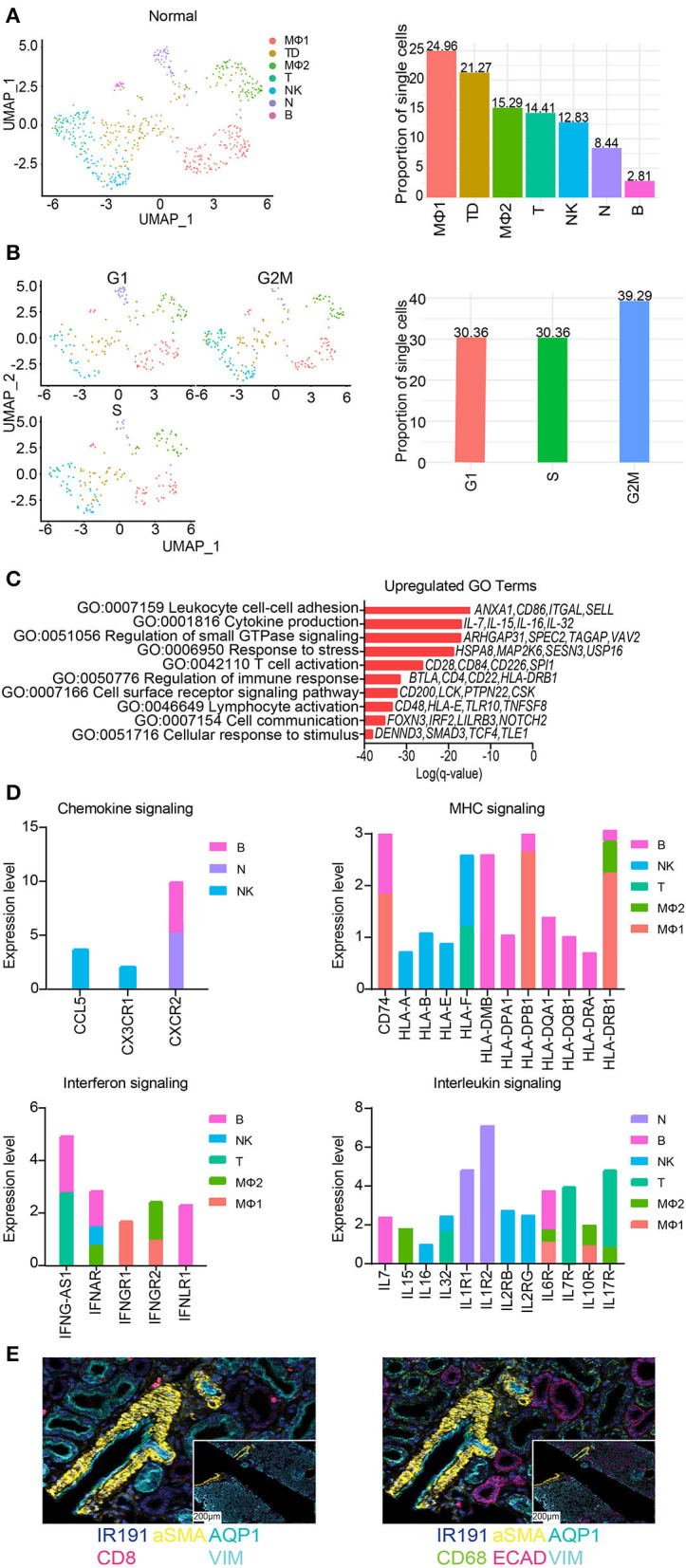
Subclustering of immune cells from normal allografts. **(A)** (Left) UMAP visualization of 7 immune subclusters. (Right) Distribution of immune cell subclusters by proportion. Clusters are labeled by color. MF, macrophage; TD, tubule doublet; MO, monocyte; T, T cell; NK, natural killer cell; N, neutrophil; B, B cell. **(B)** (Left) UMAP visualization and (Right) quantification of cell cycle phase distribution. **(C)** The top enriched Gene Ontology (GO) terms of upregulated gene signatures for immune cells. Select genes are listed. **(D)** Expression profiling of indicated pathways and cell population. FC, fold change. **(E)** Spatial morphological evaluation of allograft tissue using Imaging Mass Cytometry (IMC), (scale bar = 100 μM). ROIs are labeled numerically (scale bar = 50 μM). General tissue organization was done identifying cells expressing αSMA (smooth muscle cells), AQP1 (proximal tubule cells), ECAD (multiple epithelial tubule cells), and VIM (fibroblasts, pericytes, mesangium, and podocytes). Nuclei are also identified by IR191. Independent image channels are shown for (left) relative abundance of CD8+ T cells and (right) CD68+ macrophages.

The total number of immune cells increased from 0.25% in native kidneys to 2.04% in normal allograft kidneys (Figure 1E). We identified seven distinct subclusters of immune cells that included B (B, *FCRL*1/2^+^, *MS4A1*(*CD20*)^+^, *PAX*5^+^), macrophages 1 (MΦ1, CD68^+^, *CD*163^+^, *MRC*1^+^), macrophages 2 (MΦ2, *CD*300*E*^+^, *FCN*1^+^, *CLEC*12*A*^+^) T (T, *LEF*1^+^, *NELL*2^+^, *IL*7*R*^+^), natural killer cells (NK, *KLRF*1^+^, *GNLY*^+^, *NKG*7^+^), neutrophils (N, *IL*1*R*^+^, *CXCR*2^+^, *BTNL*8^+^) cells. The T cell cluster was comprised of T memory (*CD*2^+^, *CD*3*E*^+^, *CD*28^+^, *CD*44^+^, *CD*62*L*^+^, *CD*96^+^, *TNFSF*8^+^) and naïve T (*IL*7*R*^+^, *LEF*1^+^, *TCF*7^+^, *SARAF*^+^, *TRAT*1^+^) cell markers. We also identified a “tubule double” (TD), a technical artifact that was omitted from further analysis (57) (Figure 6A). The highest proportion of immune cells ranged from MΦ1 (24.96%), MΦ2 (15.29%), T (14.41%), NK (12.83%), and N (8.44%), B cells (2.81%) (Figure 4A). When the TD was omitted from our analyses, the total proportion of cells from most to least abundant was MΦ1 (31.70%), MΦ2 (19.42%), T (18.30%), NK (16.29%), and N (10.71%), B cells (3.57%) (Supplementary Table S6). Cell cycle analysis also revealed that most immune cells were in G2M phase (39.29%) whereas the proportion of immune cells in G1 and S phase were equal (30.36%) (Figure 6B). Immune cell markers are listed in Supplementary Table S7. Predicted transcription factors by immune cell subtype using gene expression markers are reported in the Supplementary Table S8.

Further analysis of the MΦ sub-clusters was performed. The MΦ1 cluster pathway-associated genes included signaling by chemokine signaling, receptor tyrosine kinases, leukocyte differentiation, positive regulation of cell migration. The MΦ2 cluster pathway-associated genes included PDGFRB signaling, VEGF signaling, Fc gamma receptor (FCGR) dependent phagocytosis, and signaling by interleukins.

GO analyses of normal allograft biopsies were marked by significant enrichment of cell communication, response to stress, lymphocyte activation, T cell activation, and cytokine production (Figure 6C). Congruent with our results, we also observed distinct signaling signatures including chemokines, MHC molecules, interferon-γ, and interleukin signaling (Figure 6D). Together, the described key signaling pathways are likely involved in the subclinical injury associated with the continued alloimmune response. Critically, the upregulation of proinflammatory cytokines e.g., *IL-7, IL-15, IL-16*, and *IL-32* (Figures 6C,D) was observed in normal allografts. Together, the expression of HLA-class II molecules and proinflammatory cytokines (Figure 6D) suggested that the normal allograft has some level of immune cell activation.

Imaging mass cytometry (IMC) was employed to provide spatial information and validate the presence of immune cells using a panel of antibodies. The panel of markers were applied to formalin fixed, paraffin-embedded normal allograft tissue sections. First, we observed the general tissue organization identifying cells expressing aSMA (smooth muscle cells), AQP1 (proximal tubule cells), ECAD (multiple epithelial tubule cells), and VIM (fibroblasts, pericytes, mesangium, and podocytes) (58) (top panel, Figure 6E). Nuclei are also identified by IR191. The bottom panels displayed the relative abundance of CD8^+^ T cells and CD68^+^ macrophages (Figure 6E). The normal allograft is depicted as macrophage-rich, found in areas near or around kidney cells positively expressing aSMA, AQP1, ECAD, and VIM (bottom panel, Figure 6E).

## Discussion

This proof-of-concept study represents the first interrogation of the normal functioning human kidney graft transcriptome with more than 15-months post-transplant at single cell resolution. Distinct cellular and transcriptional landscapes were identified, providing biological insights about pathways associated with normal graft function. In recent years, there has been limited improvement to long-term outcomes such that increasing the longevity of grafts is a major unmet need in kidney transplantation. We hypothesized that exploring the cells and associated transcriptome in the normal functioning graft will provide critical information to achieve and/or improve long-term function.

Our results (i) emphasized the utility of single nuclei RNA-seq in revealing the cellular heterogeneity within kidney stroma and immune cells in the kidney graft, (ii) resolved gene expression dynamics between normal native and graft kidneys, emphasizing the need to include normal allografts (instead of native kidneys) as part of control groups, and (iii) presented a comprehensive view of the kidney allograft with continuous function after 15-months-post-transplant. Herein, transcriptional changes were characterized in five cell clusters including endothelial (EC), immune (IMM), podocytes (POD), fibroblasts (FB), and proximal tubular (PT) cells which have been described to play a role in the process of injury sensing and tissue repair after transplantation. This study will serve as a foundation to further understand the cellular mechanisms of human kidney injury and reparation to restore kidney function after transplantation, which will improve tissue-based personalized therapies sought to increase graft and patient survival.

Endothelial cell (EC) heterogeneity, contributing to non-overlapping populations between the two groups, was noted as well as functional differences, due to alterations in the number of cells and the level of gene expression. EC diversity may reflect activation of different cellular states or varied EC functions including but not limited to hemostasis, leukocyte trafficking, and vascular permeability (48). Additionally, EC derived from the normal allograft were found to be dynamically involved in cell migration, proliferating, and morphogenesis. This was supported by greater EC sub-clustering and a higher cellular proportion. Our data suggest that EC are responding to both the new transplant recipient environment and injury infliction to the graft. Further proof lies in the expression of *FYN*, a member of the Src family kinases reported to be a mediator of injury and inflammation (59), in three clusters (EC1, 2, and 4). We also identified immune cell populations predicted to be phenotypically active exhibiting a proinflammatory response after transplantation in normal kidney allografts. Moreover, a unique EC cluster present only in normal allografts, EC7, was found to be enriched in toll-like receptor cascades and regulation of cellular response to stress. Likely, this EC cluster is responding to stress as consequence of some cells expressing *VCAN* and receptors attracting innate immune cells (i.e., *TLR4*). A critical finding from our evaluations was the ability of our approach to recover an important number of immune cells in both groups (with immune cells being more abundant in the normal grafts). Immune cells adapt to local microenvironments, acquiring distinct features and functional specialization. Dissecting these molecular adaptations through the evaluation at single cell transcriptome resolution in the normal graft, undergoing sustained alloimmune response injury and the effect of chronic nephrotoxic immunosuppressant drugs, promises to transform our understanding of the cell-to-cell interactions that balance response to injury and repair. Based on cell proportions, immune cell phenotypes in the normal graft are largely contributed by macrophages/monocytes, T, and B cells, which have all been described as important mediators of inflammation in human kidneys (16, 26, 60). An important finding is the predominance of CD68^+^ macrophages in the kidney allograft that is consistent with previously published reports (19, 26). In the present study, we extended this evaluation by showing that macrophages highly expressed *CD74, HLA-DPB1*, and *HLA-DRB1* (Log_2_ FC > 1.8).

Mainly, our findings emphasized the ongoing alloimmune response and associated changes in kidney parenchymal cells despite the lack of clinical evident markers of injury (normal histology, normal creatinine values) indicating the advantages of molecular approaches over standard clinical markers.

Moreover, while our cell cycle analyses estimated the cycle profiles, we also reported that the cycling immune cells were slowly dividing. As proliferation is critically linked to the quality of the immune response, the slow progression into the cell cycle protects immune cells from exhaustion and give rise to memory-like precursors, as described previously (61–63). Likewise, our results supported the presence of both T memory and naïve T cells, which may play an important role in regulating the normal allograft immune response. Taken together, these analyses reflected the diversity of the immune cell landscape and underscored the relationship between immune cells and cell cycle changes after injury occurrence.

The initial damage inflicted to the kidney allograft early in the transplant process (peri- and post-transplant) as well as the continuous low but persistent pro-inflammatory responses are denoted by the upregulated expression of injury markers in PT, the first responders of kidney injury (17, 41). In addition to *LRP2* expression*, HAVCR1*, and *VCAM1* was found to be co-expressed in the PT2 and MT clusters, suggesting that these are injured PT clusters (15, 17, 18, 42). Also, the normal allograft derived PT are exhibiting a senescence phenotype and transcriptional variance triggered by external stimuli. In response, cells changed their expression patterns to adapt to their new environment (64). This is an interesting finding as younger donor kidneys were transplanted and evaluated (mean donor age: 27.5 years old), emphasizing that senescence is a novel adaptive mechanism important for tissue repair (64, 65). Perhaps one way that these senescent cells adapt is through upregulation of metabolic pathways confirmed by DAVID analyses to promote homeostasis. Understanding how senescence of kidney tubule cells leads to normal wounding and impair repair (66) prompts further investigation.

Additionally, FB play an essential role in wound healing (67–69) and were increased in the normal allograft. It is important to note, that donor kidney grafts were younger than native kidneys. Importantly, increased expression of the activated FB cell type, and upregulation of the Wnt signaling was observed in normal grafts compared to the normal native kidneys (15, 16, 27). Thus, the relative increase in the number of FB and gene expression alterations is likely a direct consequence of injury, healing, and adaptation. Further proof is found through the ongoing alloimmune response, upregulation of proinflammatory signals by immune cells, and cellular damage exhibited by PT. Contrary to the normal native biopsies, a significant increase in *COL1A* and *PDGFRA* and slight increase in *ACTA2, DCN, PDGFRB, POSTN*, and *VIM* expression was present in normal allografts, consistent with previous observations of activation and replication of FB at wound sites (27, 67–69). The ongoing alloimmune response and FB activated at a low-level raises the possibility that these phenotypes necessitate balanced kidney repair with major cell types still retaining some level of normal function. Kidney transplant recipients continued to have normal function up to 18-months of patient follow up, indicative of resolved cellular repair. Understanding the molecular mechanisms and genes expression dynamics of the normal kidney allograft will be vital in future studies. Considering the biopsy times for the normal allografts, ranging from 15-to 24-months, these biopsies critically represented resolved injury and reparation despite some level of ongoing inflammation.

A limitation of the study is the small sample size such that the presented results cannot be overgeneralized. Evaluation of protocol biopsies from kidney grafts with normal function after 15-months post-transplant is critical as these biopsies are not collected at most of the Transplant Centers (16, 70). Moreover, to our knowledge, this is the largest published dataset of normal human biopsies kidneys (16, 24–28). Critically, the best practices for sample size determination relies on the hypothesis and number of cells (71). Our comparative study of normal v native kidney biopsies profiled 40,950 cells, which was sufficient to capture transcriptional heterogeneity, subclonal or sub-cluster populations, and generated an average of 29,645 unique genes derived from the normal biopsies.

An additional important consideration is that information concerning cell positioning is lost with snRNA-seq. Such information may be critical to properly interpret results. In an attempt to validate our results, we showed using staining approaches the normal architecture of different components of the kidney grafts. Moreover, we showed the presence of macrophages cells (CD68^+^, a heavily glycosylated glycoprotein that is highly expressed in macrophages and other mononuclear phagocytes) in proximity to injured PT and FB cells. Considering that macrophages were located near fibroblasts provided by IMC imaging, it is conceivable that macrophages coordinated repair by promoting fibroblast activation, extending upon work in the field (72, 73). Also, CD8^+^ T cells were located near PT cells; however, its role in repair is more elusive.

In conclusion, our study provides a comprehensive, transcriptional map of the normal human kidney graft using snRNA-seq. Ranging in the level of injury, endothelial, fibroblast, podocyte, epithelial, and immune cells are impacted by early inflictions to the graft (e.g., peri- and post-transplant or immunosuppression therapies) and adapt to continuous injury. These results will aid in the generation of immunomodulatory therapies to prevent and treat future kidney diseases as well as aid in the better understanding of kidney cell-cell communication for tissue repair.

## Materials and methods

### Patients and samples

Kidney graft biopsies from four kidney transplant recipients (KTRs) were studied from patients with normal/stable graft function. The Institutional Review Board approved the study, which collected surveillance biopsies, and patients signed an informed consent at time of transplantation (HP-00091954). The clinical and research activities being reported are consistent with the Principles of the Declaration of Istanbul as outlined in the “Declaration of Istanbul on Organ Trafficking and Transplant Tourism.”

Patients with normal/stable graft function were collected ≥15-months post-transplantation, had an estimated GFR (eGFR) of ≥60 mL/min/ 1.73 m^2^, no proteinuria, no circulating IgG antibodies against donor HLA at the time of biopsy, and had normal/non-specific findings in the allograft surveillance biopsies. All patients received triple-drug immunosuppression that included tacrolimus, mycophenolate mofetil, and prednisone. All patients received a standard kidney from deceased donors.

Kidney allograft tissue was obtained using an 18-gauge biopsy needle and immediately immersed in RNA*later* (Ambion). Two different pathologists performed histological evaluation.

Collection of normal native kidney biopsies (*N* = 3) were obtained as previously reported [GSE131882 (24, 25)] by a renal pathologist at different collection times. Patient samples were obtained during partial or radical nephrectomy and immediately placed in freezer storage. Patients had an estimated mean GFR (eGFR) of 62.67 mL/min/ 1.73 m^2^.

### snRNA-seq

The generation of single nuclei preparations from kidney were processed as previously described (18). Enzymatic disassociation was achieved using 2 mL of Nuclei EZ Lysis buffer (NUC-101; Sigma-Aldrich) supplemented with protease inhibitor (Sigma-5892791001) and RNase inhibitor (N2615, Promega; AM2696, Life Technologies) and incubated on ice for 5 min with 2 mL of additional lysis buffer. Nuclei were then filtered using a 40 μm cell strainer (43-50040-51; pluriSelect) and viable cells were counted using the Countess 3 Automated Cell Counter (ThermoFisher). A target of 10,000 nuclei was then sequenced on the 10x Chromium instrument (10x Genomics), generating 150 bp paired end reads.

### snRNA-seq data analyses

FastQ files generated by the 10x Genomics standard sequencing protocol were aligned to the human pre-mRNA reference sequence (build GRCh38) using CellRanger (10x Genomics, v3). The CellRanger output was used for preliminary quality control (QC). The criterion for sample inclusion were as follows: genes expressed in more than 3 cells, cells with more than 400 expressed genes, cells with <5,000 expressed genes, and samples with <2.5% mitochondrial gene expression. Samples were integrated into one dataset using the R package “Seurat” and tested for any potential batch effects due to technical differences in samples and/or cell cycle phase influencing the proliferating state of the cell. Following, cell clustering was performing using dimensionality reduction [principal component analysis (PCA) followed by uniform manifold approximation and projection (UMAP)]. Distinct cell clusters were manually annotated based on gene expression of published human cell markers (32) and expression of highly variable genes. Distinct gene expression patterns were used to annotate cluster specific markers for heightened confidence. Sub-clustering analysis of major cell populations were assessed using significant differentially expressed genes (FDR ≤ 0.05) and subsequently, used to discern enriched gene ontology terms and pathways. All expression analyses, statistical evaluations, and visualization plots (cell marker expression heatmaps and violin plots) were generated using the R Seurat software.

### Gene ontology

DAVID (Database for Annotation, Visualization and Integrated Discovery, v6.8) Bioinformatics Resource (74) and Metascape software (75) was used to map identified genes to relevant biological functions (74) and perform intra- and inter-cluster comparative analyses.

### Cell cycle analysis

The number of cells undergoing the phases of the cell cycle per cluster was performed as previously described by Tirosh et al. (76). Briefly, a core set of cell cycle phase-specific gene signatures were used to distinguished cycling from non-cycling cells. Subsequently, the average expression of each phase marker was measured and calculated using the Seurat v3 MetaFeature() function. Cells were identified as cycling [E(expression)>1 and FDR <0.05] or non-cycling (E <1 or FDR >0.05).

### Tissue biopsy staining

Hematoxylin and eosin (H&E) stained sections was performed by the Pathology Department at the University of Maryland, Baltimore using standard protocols. High resolution images were generated from formalin-fixed, paraffin-embedded tissue blocks on a Ventana Discovery Ultra Autostainer (Roche Diagnostics). Imaging mass cytometry (IMC) was performed at the Histopathology and Tissue Shared Resources Core of Georgetown University also from formalin-fixed, paraffin-embedded tissue blocks. Marker panels were ordered from Fluidigm (Standard Biotool) which included ALPHA SMOOTH MUSCLE ACTIN (aSMA, catalog #31410117D), CD8A (catalog #3162034D), COLLAGEN 1A (COL1A1, catalog #3169023D), E-CADHERIN (ECAD, catalog #3158029D), and VIMENTIN (VIM, catalog #3143027D). AQUAPORIN 1 (AQP1) was purchased from ABCAM (catalog #AB178353). Images were obtained using the Fluidigm Hyperion Imaging System with appropriate fluorescence filters and processed using the Quantitative Pathology and Bioimage Analysis (QuPath) Software v0.3.2 (77). Control experiments were performed and yielded no observable non-specific staining. All patient samples were de-identified prior to imaging.

### Transcription factor analyses

Gprofiler (78), a web-based tool, was used to find the predicted transcription factors associated with the immune cell expression data.

## Data availability statement

The datasets presented in this study can be found in online repositories. The names of the repository/repositories and accession number(s) can be found below: https://www.ncbi.nlm.nih.gov/geo/, GSE195718.

## Ethics statement

The studies involving human participants were reviewed and approved by the Institutional Review Board approved the study, which collected surveillance biopsies, and patients signed an informed consent at time of transplantation (HP-00091954). The clinical and research activities being reported are consistent with the Principles of the Declaration of Istanbul as outlined in the Declaration of Istanbul on Organ Trafficking and Transplant Tourism. The patients/participants provided their written informed consent to participate in this study.

## Author contributions

Conceived and designed the analysis and collected the data: JM, AS, DM, and VM. Participated in research design, sample procurement, and performance of the research: DM and VM. Contributed to analytical tools: AS. Performed the analysis: JM, AS, and VM. Wrote the paper: JM, AS, EB, TR, and DM. All authors contributed to the article and approved of the submitted version.

## Funding

This research reported in this publication was supported by the National Institute of Diabetes and Digestive and Kidney Diseases (NIDDK) of the National Institutes of Health under awards numbers: R21DK100678 (DM) and R01DK109581, R01DK122682 (VM), and 3R01DK122682-03S1 (JM).

## Conflict of interest

The authors declare that the research was conducted in the absence of any commercial or financial relationships that could be construed as a potential conflict of interest.

## Publisher's note

All claims expressed in this article are solely those of the authors and do not necessarily represent those of their affiliated organizations, or those of the publisher, the editors and the reviewers. Any product that may be evaluated in this article, or claim that may be made by its manufacturer, is not guaranteed or endorsed by the publisher.
