# Supplementary material for: The cellular landscape of the normal kidney allograft: Main players balancing the alloimmune response
Source: Front Transplant. 2022 Oct 17;1:988238. doi: 10.3389/frtra.2022.988238 (PMC11235379; doi:10.3389/frtra.2022.988238)
Supplement: Supplementary file 1 [file Table_1.docx]

**Table S1. Additional patient samples demographics.**

|  | Normal native | Normal allograft |
| --- | --- | --- |
| Mean donor age | 59.00 | 27.50 |
| Mean recipient age | - | 51.00 |
| Mean sCR | 1.13 | 0.89 |
| Mean time of collection | - | 18.00 |

Age denoted in years; sCR, serum creatinine levels denoted in mg/dL; time of collection denoted in months

**Table S2. Normal PT1 upregulated metabolic pathways identified by KEGG pathway analysis.**

| Pathway | *p*-value | Adjusted  *p*-value | Combined Score | Genes |
| --- | --- | --- | --- | --- |
| Phosphatidylinositol signaling system | 1.99E-10 | 3.88E-08 | 629.25 | *DGKB DGKH IMPA2 INPP5B MTMR3 PIK3CB PIP5K1A PLCB1 PLCG2* |
| Inositol phosphate metabolism | 5.52E-10 | 5.38E-08 | 713.58 | *ALDH6A1 IMPA2 INPP5B MTMR3 PIK3CB PIP5K1A PLCB1 PLCG* |
| Beta-alanine metabolism | 2.01E-09 | 1.31E-07 | 1328.07 | *ABAT ALDH2 ALDH6A1 ALDH7A1 DPYS HIBCH* |
| Glycerolipid metabolism | 5.02E-09 | 2.45E-07 | 665.01 | *AGPAT3 ALDH2 ALDH7A1 DGKB DGKH PLPP1 PNPLA3* |
| Lysine degradation | 6.32E-09 | 2.47E-07 | 633.44 | *ALDH2 ALDH7A1 BBOX1 EHMT1 MECOM*  *NSD1 PHYKPL* |
| Phospholipase D signaling pathway | 8.64E-09 | 2.81E-07 | 330.27 | *AGPAT3 DGKB DGKH PIK3CB PIP5K1A PLCG2 PLD1 PLPP1* |
| Tryptophan metabolism | 1.71E-08 | 4.75E-07 | 790.29 | *ACMSD AFMID ALDH2 ALDH7A1 ALDH8A1 KMO* |
| Sphingolipid signaling pathway | 5.34E-07 | 1.30E-05 | 241.62 | *CERS4 CERS6 PIK3CB PLD1 SGPP1 SPTLC3* |
| Purine metabolism | 9.22E-07 | 2.00E-05 | 213.31 | *ADK AK2 ENTPD5 NME7 PDE1A PDE7B PDE10A* |

**Table S3. Tubule cluster cell senescence signature.**

| Gene | Avg Log_2_ FC | Adjusted *p*-value | Cell type |
| --- | --- | --- | --- |
| *CALM2* | 5.738 | 2.05E-135 | MT |
| *CAPN2* | 0.758 | 4.98E-01 | MT |
| *HIPK2*  *HIPK2*  *HIPK2* | 0.610 | 5.11E-36 | PT1 |
|  | 0.839 | 2.13E-03 | PT2 |
|  | 0.635 | 2.35E-03 | MT |
| *MAPK14* | 0.681 | 1.53E-10 | PT1 |
| *NFATC3* | 0.564 | 5.10E-13 | PT1 |
| *NFATC3* | 0.793 | 2.73E-02 | MT |
| *NFATC3* | 1.326 | 2.26E-03 | PT2 |
| *PPP3R1* | 5.468 | 1.26E-118 | MT |
| *PPP3R1* | 5.320 | 1.73E-27 | PT2 |
| *RAD50* | 6.265 | 2.97E-279 | PT1 |
| *RAD50* | 6.089 | 3.26E-179 | MT |
| *RAD50* | 6.305 | 5.15E-47 | PT2 |
| *RAD9A* | 0.477 | 1.68E-06 | PT1 |
| *RAD9A* | 1.064 | 1.14E-09 | MT |
| *SMAD3* | 0.730 | 8.65E-29 | PT1 |
| *SMAD3* | 0.602 | 7.13E-07 | MT |
| *SQSTM1* | 1.460 | 5.81E-62 | PT1 |
| *SQSTM1* | 1.188 | 2.39E-08 | MT |
| *SQSTM1* | 1.136 | 1.92E-02 | PT2 |

Avg Log_2_ FC, Average Log_2_ fold change; MT, mixed tubule; PT1, proximal tubule 1; and PT2, proximal tubule 2.

**Table S4. Select endothelial cell markers derived from the normal native biopsy.**

| Cluster | Cluster-specific cell markers |
| --- | --- |
| EC1 | *APPBP2 ATP9B AUTS2 BDP1 CENATAC CHD6 DCAF8 DDX17 DHRS3 DNAJC3 EFCAB13 EGFL7 ENG ENSG00000241962 EP400 ERICH1 FLT4 GPATCH2L HDLBP HFM1 HNRNPD HSPA1B HSPG2 INO80 LIFR LINGO1 LRMDA MARK4 ORC4 OXNAD1 PBRM1 PCNX4 PDE10A PIGN PRPF3 PSMD1 RAMP3 RAP1GDS1 RBPJ RYR3 SEC62 SNX6 SPG7 SUPT20H SYCP2L TASOR TBCD TNS2 TRIM24 TTC14 UBR1 USP8 VPS35L ZNF75D ZNF782* |
| EC2 | *RCAN2 EMCN GRIP2 RGL1 KHDRBS3 LDB2 SH3KBP1 ENSG00000284686 SPRED2 DYSF CHRM3 ANO6 CDH13 PLPP3 RASGRP3 RPRD1A DNAJC1 PBX1 RIN2 AR ITGA6 JAM3 XIST RAPGEF4 NCALD DENND4C MAML3 HMBOX1 PREX2 SEMA6A MEIS2 FYN ZEB1 YES1 NOSTRIN NRP1 DOCK4 PTPRB SEC14L1 KLF7 RASAL2 SRGAP2 EPAS1 GRB10 ETS1 APP SPTBN1 CCNY SMYD3 SYNE2 TEK GNAQ* |
| EC3 | *ABI3BP ADAMTS1 ADAMTS6 ADD1 AKAP13 ATP8B1 COL8A1 CRYBG3 CSRNP1 DYNC2H1 EEF2K ELN EPHA4 EXT1 FBN1 FBXL17 FGF2 FLNB FN1 FRY FUT8 GLUL HDGFL3 HSPB1 IL33 ITGAV ITSN2 JAG1 KIF13B KLF2 MAP1B MAP2 MAP4 MBNL1 MGP NAV2 PDCD4 PDGFD PECAM1 PLCG2 PLXNA2 PLXNA4 PRKX PTPN14 RAPGEF1 RPS6KA2 SMAD6 SMAD7 SPEF2 SRGN ST8SIA6 STK24 SULF1 SYBU SYNJ2 TANC2 TIPARP TM4SF1 TSPAN2 UTRN VEGFC VIM WNT5B WWTR1 ZDHHC21* |
| EC4 | *BCL3 EMP1 ENSG00000267519 FOS FOSB GPSM3 LINC00486 RASGEF1B SOCS3 ZFP36* |
| EC5 | *ACSL5 AKAP12 ARHGAP26 ARRDC3 ATP2B4 B2M BDKRB2 BMP6 CALCRL CD9 CHN1 CMKLR1 CTNNB1 DLL1 DNAJA1 DUSP5 ECT2 ENSG00000282278 FGFR1 FLT1 FRMD8 GATA6 GNA12 HERPUD1 HES1 HIPK3 HLA-DRA IKBKB ILK INPP5D IRAK3 JCAD KALRN KDM5D LDLRAD4 LPL LRRK1 MN1 NACC2 NFATC2 NFKB1 NOVA2 NUAK1 OSMR P2RY8 PPP3CA PRKCE PXDN RIN3 RPH3AL RPS12 SASH1 SH2B3 SH3RF3 STARD13 TGFBR3 TLE2 TMEM33 TNFAIP1 TNFRSF1A USP13 ZNFX1* |
| EC6 | *ADAM9 ADCY1 ATAD2 BCAR3 CALCOCO2 CD74 CD99 COX17 DAAM2 DAZAP2 EDNRB EGFR EMP2 ETS2 FRMD4A GJA5 GNAI2 HIVEP3 HLA-F HMGN3 HTR2C IL1RL1 IRF1 ITGA1 KDR LMO2 MAP2K6 MAPRE1 MED10 NBN NDRG4 NEAT1 NEDD4 NID1 NOTCH1 PGR PPARG PTGIS PTK2B RIPOR2 RPLP1 RPS6 RRAGB RREB1 SERINC3 SLC26A5 SLCO3A1 SLF1 SMAD9 SMARCA2 TAOK2 TBC1D20 TBX3 TPM1 TRIM35 TTC5 UNC5B WNK3 ZFHX3* |

**Table S5. Select endothelial cell markers derived from the normal allograft.**

| Cluster | Cluster-specific cell markers |
| --- | --- |
| EC1 | *ACACA AFF1 AJAP1 APPL2 ARGLU1 ARHGAP10 ARID1A ARIH1 ASCC3 ATE1 ATF7IP2 B4GALT1 BCL2L1 BTN3A1 BTN3A2 C20ORF194 CALCOCO2 CASKIN2 CCND1 CD9 CDK6 CEP350 CFLAR CREBBP DDX5 DYNC1LI2 EGFR ERGIC1 FAM111A FAM120B FKBP5 GPR107 GRAMD2B HLADPB1 IL18R1 IL4R INPP5A INPP5K KAT6A KCNMB4 KHDRBS3 KL LATS1 LRRC8B MACF1 MAP2K1 MAU2 MLXIP MORC3 MTOR MXD4 MYO6 NAP1L1 NBAS NCOA3 NDEL1 PAPSS2 PER1 PITPNA PLA2G4C PPM1B PRX RABGAP1L RFTN2 RGS12 RHOT1 RNF115 RUSC2 SCLT1 SH3PXD2A SHROOM2 SHROOM4 SPARC SPRY4 SRSF5 STAM2 STARD13 STK39 TAL1 TBC1D8 TMEM44 TMEM51 TRAK2 TRIB2 UNC13B VIPR1 WASF2 XRRA1* |
| EC2 | *ANKRD36C ARFGEF1 ASAP2 ASIC2 BMP6 CAPZB CASK CBL CCM2 CDC42BPB CHD6 CMIP CTBP2 CTIF CYTH1 DENND6A DOCK6 ECHDC2 FAM120A GSE1 HNRNPDL HNRNPH1 HSPG2 INO80D ITGB5 KHDRBS1 KIRREL1 KSR1 MPRIP MYO10 NFASC OPHN1 PDE2A PHACTR1 PHC2 PITPNC1 PITPNM2 PRAG1 PRKAR2A PRKD2 PRKRIP1 PRRC2B RAMP3 REV1 RFTN1 RILPL1 RIN3 SFMBT2 SP3 SPG7 ST3GAL2 STAG2 STRN3 SUSD6 SYCP2L TMEM131L* |
| EC3 | *ABL1 ACTN4 AIG1 AKAP9 ARSB ATP1B1 ATP9B ATRN BOD1L1 CABIN1 CBR4 CCAR1 CLEC16A CPLANE1 CTDSPL2 DDX46 EP300 EP400 FBXL5 FNBP4 FOXK2 GCC2 HERC2 HP1BP3 IGF2BP2 IGF2R KIF1B KLHL20 LARP1 LUZP1 LYPLAL1 MINK1 MPHOSPH8 NPLOC4 NSRP1 PAN3 PRKDC RBM25 RPTOR SCAF11 SCFD2 SEC63 SH3RF1 SIPA1L1 SKI SLC44A2 SMARCC1 SPAG16 SPAG9 SREK1 SRRM1 STXBP5 TBC1D19 TCF25 TET2 TLK1 TRIM44 USP47 VPS53* |
| EC4 | *ACBD6 ADAMTS1 AKAP10 ANKFY1 AP1G1 ARID2 ARID5B ARNT BACH1 BCL9 BEND7 CALCR CAND1 CCNL1 CDC42 CDC73 CHFR CNTN5 COQ8A CSDE1 CTNNAL1 DCAF10 DCP1A DHX29 DISP1 DTX3L EIF3H ENOX2 EXT2 FAM126B FAM222B FBXO25 FIP1L1 FNDC3A FRS2 GNG7 GPBP1 GPC5 HDAC4 HELZ HEXA IFI27 IFIH1 IFIT1 IRF9 IRS1 KDM4C KIDINS220 KLHDC10 KMT5B LAP3 LPAR6 MAP3K5 MARK3 MAVS MTFR1 MX2 NCOA1 NDFIP1 NDUFS1 NLGN4Y NLRC5 NRF1 NRG2 NUMB PCBP2 PHF11 PICALM PLEKHO1 PPP2R5E PSMD1 PTBP1 RAB5A RCOR3 RFFL RIPK1 RNF111 RSAD2 S100Z SBF2 SEC24B SMG6 SMURF1 SP110 ST7L STAT2 STAU1 SYNRG TAPBP TBC1D15 TBC1D2B TBC1D5 THRB TPP2 TRAPPC10 TRIM22 TUBGCP3 UBAC2 UBE2J1 UBE4B UBR1 UMAD1 XRN1 ZFAND3* |
| EC5 | *AAK1 ABCB1 ABCC4 ACTB ACTR2 ADK ALPK1 ASS1 ATL2 ATP2A3 ATP8A1 CDK14 CDK19 CHD3 CLK4 CMPK1 DDX39B DYNC1H1 EEF1A1 EEF2K EEFSEC ENTPD4 EPHA3 FGF2 GCH1 GLUD1 GTF2F2 ITGA2 KIF13B LRRK2 MAP3K11 MAP3K14 MAP4K3 MAST2 MAST4 MERTK MET NAV2 NEK3 NMNAT3 PAFAH1B1 PDK3 PRKACB RALB RAPGEF3 SERPINE2 SMC6 STK17A STK38L SUCLA2 SUCLG2 SULF1 TAOK1 TGFBR1 TTLL7 UBE2G1 ULK4 VCP VRK2 ZRANB3* |
| EC6 | *ADAMTS12 ADGRA2 AKAP12 ANGPT2 BCAR1 CARD10 CCDC141 CCL21 CD200 CD47 CORO1C CTSH DDR2 DMTN DNM1L DOCK8 DPP4 DPYSL3 EPHB1 F11R FGF13 GCNT2 GNA12 GPR173 HACE1 IGF1 INPP5F ITGB1BP1 ITGB3 ITGB8 JMY KANK2 KIF2A LYVE1 MARK1 MGAT5 MITF MYO18A MYSM1 NTN1 PALLD PDCD6 PDE4B PDGFC PIK3C2G PLEKHG3 PRKCZ PRKG1 PROX1 RELN RERE ROCK2 SEMA3A SEMA6D SIRPA SRGAP3 TBX1 TNFRSF11A TNS1 TSPAN11 YTHDF3* |
| EC7 | *ABCA1 BAZ2A C18ORF25 CAMKMT CCNI COL3A1 DAB2 DCAF5 DCUN1D4 DIPK2A DOCK7 FANCL GALNT15 GFRA1 GTF2H1 HSP90B1 HYAL2 KDM5D LYPLA1 MAN1A2 MAN1C1 MECP2 MFHAS1 MINDY4 MORF4L2 MTMR2 NAA25 NEURL1B NFE2L2 OSBPL8 OTUD4 PAXBP1 PHF8 PIK3C3 PKD2 PLOD2 PPP2CA PRPF4B RACK1 RIOK3 RNF125 RNF149 RNF169 RNF180 RNF216 RNF38 RNF4 SKP1 TLR4 TMTC2 TNFRSF1A TPD52L1 TRERF1 TRIM27 TTLL5 UBE2E3 UBE3D USP25* |

**Table S6. Total number and proportion of single nuclei derived from immune cells of the normal allograft.**

|  | Cell cluster | Total number of cells | Proportion of total cells (%) |
| --- | --- | --- | --- |
| 1 | Macrophage 1 (MΦ1) | 142 | 31.70 |
| 2 | Tubule doublet (TD) | 121 | 21.27 |
| 3 | Macrophage 2 (MΦ2) | 87 | 19.42 |
| 4 | T (T) | 82 | 18.30 |
| 5 | Natural killer (NK) | 73 | 16.29 |
| 6 | Neutrophil (N) | 48 | 10.71 |
| 7 | B (B) | 16 | 3.57 |
|  | Total | 488* | 100.00* |

*****TD contribution was omitted from analysis

**Table S7. Select immune cell markers derived from the normal allograft.**

| Cluster | Cluster-specific cell markers |
| --- | --- |
| B | *AIDA AIM2 AKAP13 AKIRIN1 ALOX5 AMFR ARHGAP17 ARHGAP24 ARHGAP31 ARHGEF18 ATP2B1 AUTS2 BANK1 BCAR3 BCL2 BICD1 BIRC3 BLK BLNK BMP2K BRAP BTK BTLA C1RL CAMK1D CASK CBY1 CCND3 CD180 CD200 CD22 CD40 CD74 CDK14 CDK5RAP3 CLOCK CNKSR2 CNR2 CSNK1G1 CSNK1G3 CYBA CYFIP2 DENND4A DGKD DGKI DLGAP4 DNAJB6 DTX1 EIF2AK1 EIF2AK3 ELF1 ELP2 ERP29 EZR FBXW7 FCRL3 FER FGD2 FKTN FUS FUT8 G3BP1 GEN1 GKAP1 GNA12 GNAS GNG7 HDAC6 HERC4 HINT1 HLA-DMB HLA-DPB1 HLA-DRA HLA-DRB1 HMGB2 IFNLR1 IGHG1 IGHM IL7 INPP5D INSIG2 IQSEC1 ITPR1 KCNN4 KDM4C KLHL6 LAMTOR4 LBH LCMT1 LIMD1 LRIG2 LY86 MAP2K3 MAP3K1 MAP4K4 MAPRE2 MBTD1 MEF2C MGAT5 MICB MS4A1 MTPN NCOA3 NDST1 NEK6 NEPRO NFKB1 NOTCH2 NRG1 NUCKS1 OTUD5 P2RX5 PARP1 PBXIP1 PCID2 PHF14 PIK3AP1 PIP5K1B PJA2 PLCG2 PLEKHG1 PLPP3 PPP1R16B PPP2CA PPP3CA PPP3CB PRAG1 PRKCB PRKCE PSMB1 PSMB7 PTK2 PTK2B PTPN2 PWP1 RAC2 RALGPS2 RBM14 RBX1 REL RFWD3 RHBDF2 RHOH RIPOR1 RIPOR2 RNF43 SCIMP SEMA4B SESN3 SH2B2 SHANK3 SHOC2 SIPA1L3 SLAMF6 SLC15A2 SLC15A4 SLC38A9 SNX13 SNX25 SP100 SPPL2B SQSTM1 SREBF2 ST6GAL1 STAP1 STX7 SWAP70 SYK TAGAP TAPBP TLE1 TLE4 TLR10 TM2D3 TNFRSF13B TNFRSF13C TRAF3 TRAF5 TRIM13 TRIO TUBD1 TWSG1 UCP2 URI1 VAV2* |
| MФ1 | *ABCA1 ABCC4 ABCC5 ALOX5 ANKS1A ANO10 AP1B1 AP2A2 APPL2 ARHGAP18 ARHGAP21 ARHGAP24 ARRB1 ARSB ATG7 ATP6V0A1 ATP8B4 ATRN AUTS2 BMP2K BNIP3L CAMK1D CCDC88A CD163 CD163L1 CD4 CD74 CHKA CMKLR1 CNTLN CSF1R CYBB CYFIP1 DAPK1 DENND1A DISC1 DISP1 DNAJC13 DOCK2 DOCK4 DST ELMO1 EPB41L2 EPB41L3 EPS8 FCGRT FCHO2 FCHSD2 FER FGF13 FKBP15 FMNL2 FMNL3 FOXO3 FRMD4A FTX GAB1 GAB2 GAS6 GCNT2 GPR155 GRK3 GSN HDAC9 HLADRB1 IFNGR2 IL10RA ITGA9 ITGB5 ITPR2 ITSN1 JAK2 KCNMA1 KCNQ3 KIF16B LDLRAD4 LRP1 MAN1A1 MCTP1 MEF2A MEF2C MERTK MITF MRC1 MSR1 MVB12B MYO1E MYO5A NBEA NRIP1 NRP1 OSBPL11 OSBPL1A OSBPL3 PACS2 PAK1 PDGFC PEAK1 PHACTR1 PID1 PLCG2 PLXND1 PPARD PSAP QKI RAB31 RHOQ RREB1 RUFY1 RUFY3 RYR1 SCARB1 SDCCAG8 SGK3 SHTN1 SIGLEC1 SLC1A3 SLC23A2 SLC2A13 SLC2A9 SLC45A4 SLC4A7 SLC8A1 SLC9A9 SLCO2B1 SNX24 SNX30 SNX6 SPI1 SPIRE1 SPRED1 SRGAP1 SRGAP2 SRGAP2B SRGAP2C SRGAP3 STAB1 STARD13 STK3 STON2 STX7 SYK TBC1D14 TBC1D2B TBC1D4 TIAM1 TLR2 TNFAIP2 TPCN1 TRPM2 TTBK2 TTN UVRAG VPS13C WLS* |
| MФ2 | *ADAM10 ADGRE2 AHR AIF1 AP3B1 BCR CBL CD300A CD300LF CD4 CD44 CD46 CD48 CD55 CD74 CD86 CLEC7A CMTM7 CR1 CSK CTNNB1 CTSC DCLRE1C DOCK11 DOCK2 DOCK8 ELF4 EP300 FCER1G FGL2 FGR FKBP1A FOXP1 FYN GAB2 GPR137B HDAC9 HLA-DRB1 IFNGR1 IFNGR2 IL15 IL6R IRF1 ITGA4 ITGAL ITGAM ITGB2 JAK2 JAML KLF6 LCP1 LGALS9 LILRB2 LRRK2 LST1 LTBR LYN MAP3K8 MEF2C MNDA MSN NCKAP1L NDRG1 NFAM1 NFKBIZ NOTCH2 P2RX7 PAG1 PDPK1 PELI1 PKN1 PLSCR1 POU2F2 PPP2R3C PRAM1 PRKCB PRKCD PRKCE PSEN1 PTK2B PTPN6 PTPRC PTPRJ RARA RC3H1 RC3H2 RHOA RIPOR2 SH3RF1 SIRPB1 SKAP2 SLC11A1 SNX27 SOS2 SP3 SPI1 STAT3 STAT6 STXBP2 SUPT6H SWAP70 SYK TCIRG1 THEMIS2 TLR1 TLR2 TMEM131L TNFRSF14 TNFRSF1B TSC1 TYROBP VAV1 VSIR WAS ZFP36L2* |
| N | *ABHD5 ACTN1 ADAMTSL4-AS1 AGTPBP1 ALPK1 ANXA11 AQP9 ARHGAP26 BASP1 BAZ2B BCL6 BICDL1 BTNL8 CACNA1D CACNB4 CALD1 CAMK1D CASR CCSER1 CFLAR COP1 CPQ CR1 CREB5 CSF3R CXCR2 CYSTM1 DNAH14 DOCK5 DYSF ECE1 ENOX1 EPHB1 EPS8L2 ERBB4 ESRRB ESRRG FAM126B FAM49B FCGR2A FNBP1L FNDC3B GAB2 GCA GK GPC5 GPHN HCK HOXD3 IGF2R IL17RA IL1R1 IL1R2 IL6R JAML KCNJ15 KCTD1 KIAA0825 KIF13A LILRB3 LIMK2 LITAF LRRK2 LYN LYST MAGI2 MBOAT7 MECOM MGAM MMP25 MNDA MXD1 MYO1F NAMPT NCF1 NCF2 NDEL1 NEAT1 NIBAN1 NUMB PHF21A PKHD1 PLCL1 PLXNC1 PREX1 PTEN PTH2R PTPRJ RAB11FIP1 RAB31 RBM47 RESF1 RFX2 RGL3 RIPOR2 RNF24 SBF2 SCNN1A SEC14L1 SGK2 SLC11A1 SLC25A37 SLC8A1 SMAP2 SMCHD1 SORL1 SRPK2 SSH2 STX3 SVIL TBL1X TECPR2 TMEM154 TNFRSF10C TRIM25 USP15 USP32 VMP1 XPO6* |
| NK | *ABI1 ACTR2 ACTR3 ADAM10 ADCY7 ADD1 ADGRE5 ANKRD17 ANO6 ANXA1 AP1G1 APBB1IP APOBEC3G ATM B2M BCL11B BRAF BTN3A1 BTN3A2 BTN3A3 CARD11 CASP8 CBLB CCDC92 CCL5 CCND3 CD2 CD226 CD244 CD247 CD300A CD38 CD47 CD48 CD6 CD96 CD99 CDC73 CDK6 CNOT4 CRACR2A CTSC CUL4A CX3CR1 CYBA DCAF1 DCLRE1C DENND1B DGKZ DHX36 DOCK10 DOCK11 DPP8 EPG5 ERAP2 ERBIN ETS1 FAM49B FBXW7 FCGR3A FCMR FCRL3 FYN GAB3 GBP2 GBP5 GNLY GPR174 HAVCR2 HERC6 HIPK2 HLA-A HLA-B HLA-E HLA-F IFI16 IFNAR2 IKZF1 IKZF3 IL16 IL18RAP IL2RB IL2RG IL32 INPP5D IRAK4 IREB2 IRF1 ITCH ITGA4 ITGA6 ITGAL ITGAM ITGB1 ITGB2 ITK ITPKB JAK1 KIF5B KLF13 KLF6 KLRC2 KLRC3 KLRC4 KLRD1 KLRG1 KLRK1 LCK LCP1 LCP2 LNPEP LPXN LSM14A MAP3K8 MAPK1 MBP MCU MFSD6 MLLT3 MSN MYO1G NCR1 NFATC2 NKG7 NLRC3 NLRC5 OGT OPTN PDGFD PIK3R1 PIP4K2A PLCB1 PLCL2 PLEK PLEKHA1 PPP3CA PRF1 PRKCH PRKCQ PRKD2 PRKDC PRKX PTGDR PTK2B PTPN22 PTPRC PYHIN1 RAB27A RAB29 RAB6A RAB8B RAC2 RAP1GAP2 RASAL3 RASGRP1 RFTN1 RHOH RIN3 RIPK1 RIPOR2 RNF125 ROCK1 RORA RTN4 RUNX1 RUNX3 SASH3 SEMA4D SENP7 SERPINB9 SH2D1B SH3KBP1 SKAP1 SLA SLAMF6 SLC39A10 SNRK SOS1 SP3 SPON2 SRP54 SRPK2 SSBP3 STAT3 STK10 STK39 STK4 STX8 TAPBP TBC1D10C TBK1 TENT2 TGFBR1 TGFBR3 THEMIS TMBIM6 TOX TRIM22 TRIM38 TSPAN32 TXK UBASH3B VAV3 WDR7 XRCC5 YES1 ZAP70 ZBTB1* |
| T | *ADAM17 AMBRA1 ATG5 B2M BCL11B BCL2 BTN3A1 CAMK4 CARD11 CASP8 CBLB CCND3 CD2 CD28 CD3E CD44 CD47 CD6 CD84 CDK6 CORO1A CRACR2A CTNNB1 FOXP1 FYN GLMN HLA-A IL6ST IL7R ITGAL ITK ITPKB JAK3 LCK LCP1 LEF1 LY9 MALT1 MTOR MYH9 NCK1 NCK2 NLRC3 PAG1 PPP3CB PRKCQ PRKCZ PRKDC PTPN11 PTPN22 PTPRC RAB27A RASAL3 RASGRP1 RC3H2 RHOH RIPOR2 RORA RUNX1 RUNX2 RUNX3 SLAMF1 SLAMF6 SMAD3 STAT5B TCF7 TESPA1 TGFBR2 THEMIS TMEM131L TNFSF8 TOX ZAP70* |

**Table S8. Top 10 predicted transcription factors for immune cell subclusters.**

| Cluster | Transcription factors (TFs) | Adjusted *p-*value |
| --- | --- | --- |
| B | E2F-2; motif: GCGCGCGCGYW | 1.09E-12 |
|  | E2F-4; motif: SNGGGCGGGAANN; match class: 1 | 1.87E-12 |
|  | ZF5; motif: GSGCGCGR; match class: 1 | 2.42E-12 |
|  | ZF5; motif: GGSGCGCGS; match class: 1 | 2.48E-12 |
|  | E2F-2; motif: GCGCGCGCGYW; match class: 1 | 1.03E-11 |
|  | ETF; motif: GVGGMGG | 8.19E-11 |
|  | ETF; motif: GVGGMGG; match class: 1 | 9.70E-11 |
|  | E2F-1; motif: NNNSSCGCSAANN | 4.45E-10 |
|  | E2F4; motif: YCCCGCCNCNNSSNNSNN; match class: 1 | 5.95E-10 |
|  | E2F-2; motif: GCGCGCGCNCS; match class: 1 | 9.87E-10 |
| MФ1 | EGR; motif: CGCCCCCGCNN; match class: 1 | 5.58E-08 |
|  | EGR-1; motif: GCGGGGGCGG; match class: 1 | 6.59E-08 |
|  | EGR-1; motif: GCGGGGGCGG | 8.17E-08 |
|  | EGR-1; motif: GCGGGGGCGG | 2.07E-07 |
|  | WT1; motif: CGCCCCCNCN; match class: 1 | 2.12E-07 |
|  | ETF; motif: GVGGMGG; match class: 1 | 2.50E-07 |
|  | KROX; motif: CCCGCCCCCRCCCC | 5.85E-07 |
|  | EGR1; motif: NCNCCGCCCCCGCN | 1.32E-06 |
|  | Egr-1; motif: GCGGGGGCGG; match class: 1 | 1.65E-06 |
|  | EGR; motif: CGCCCCCGCNN | 3.53E-06 |
| MФ2 | ETF; motif: GVGGMGG; match class: 1 | 6.42E-25 |
|  | ZXDL; motif: GSGSCNNGGGMRGCNCCGGGS; match class: 1 | 4.75E-23 |
|  | E2F-3; motif: GGCGGGN; match class: 1 | 1.55E-22 |
|  | ETF; motif: GVGGMGG | 7.41E-22 |
|  | E2F; motif: GGCGSG; match class: 1 | 3.47E-20 |
|  | MAZ; motif: GGGMGGGGSSGGGGGGGGGGGG; match class: 1 | 4.04E-18 |
|  | SP4; motif: NNGNARGRGGCGGRGCNNRR | 4.17E-18 |
|  | E2F-2; motif: GCGCGCGCNCS; match class: 1 | 5.33E-18 |
|  | Churchill; motif: CGGGNN; match class: 1 | 1.13E-17 |
|  | ZXDL; motif: GSGSCNNGGGMRGCNCCGGGS | 3.72E-17 |
| N | None |  |
| NK | ETF; motif: GVGGMGG; match class: 1 | 2.62E-17 |
|  | ETF; motif: GVGGMGG | 6.70E-13 |
|  | RNF96; motif: BCCCGCRGCC; match class: 1 | 6.84E-13 |
|  | EGR-1; motif: GCGCATGCG; match class: 1 | 1.44E-10 |
|  | TAFII250; motif: RARRWGGCGGMGGNGR; match class: 1 | 2.83E-10 |
|  | EGR -1; motif: GCGCATGCG | 3.73E-09 |
|  | WT1; motif: SMCNCCNSC; match class: 1 | 3.90E-09 |
|  | RNF96; motif: BCCCGCRGCC | 1.02E-08 |
|  | ZF5; motif: NRNGNGCGCGCWN; match class: 1 | 1.72E-08 |
|  | MAZ; motif: GGGMGGGGSSGGGGGGGGGGGG; match class: 1 | 3.97E-08 |
| T | ETF; motif: GVGGMGG; match class: 1 | 8.08E-25 |
|  | ETF; motif: GVGGMGG | 1.02E-22 |
|  | E2F-2; motif: GCGCGCGCGYW | 3.97E-16 |
|  | E2F-1; motif: NGGGCGGGARV; match class: 1 | 1.77E-15 |
|  | E2F-2; motif: GCGCGCGCGYW; match class: 1 | 5.73E-15 |
|  | TAFII250; motif: RARRWGGCGGMGGNGR; match class: 1 | 3.48E-14 |
|  | E2F-1; motif: NNNSSCGCSAANN; match class: 1 | 6.32E-14 |
|  | TCF-1; motif: ACATCGRGRCGCTGW; match class: 1 | 1.36E-13 |
|  | RNF96; motif: BCCCGCRGCC | 1.73E-12 |
|  | E2F-4; motif: NTTTCSCGCC; match class: 1 | 2.39E-12 |
